# The Association Between Food Insecurity and Adverse Health Outcomes in Children and Adolescents: An Umbrella Review

**DOI:** 10.1002/fsn3.71625

**Published:** 2026-04-12

**Authors:** Seyedeh Parisa Moosavian, Farhang Hameed Awlqadr, Sanaz Mehrabani, Kimia Mazinani, Faramarz Jalili, Felicity MacIsaac, Seyed Mojtaba Ghoreishy, Behzad Ebrahimi, Mohammad Ali Hojjati Kermani, Sajjad Moradi

**Affiliations:** ^1^ Department of Community Nutrition, Vice‐Chancellery for Health Shiraz University of Medical Sciences Shiraz Iran; ^2^ Department of Food Science and Quality Control, Halabja Technical College, Sulaimani Polytechnic University Sulaymaniyah Iraq; ^3^ Isfahan Cardiovascular Research Center, Cardiovascular Research Institute Isfahan University of Medical Sciences Isfahan Iran; ^4^ Research Center for Evidence‐Based Health Management Maragheh University of Medical Sciences Maragheh Iran; ^5^ Department of Nutrition and Food Science Maragheh University of Medical Sciences Maragheh Iran; ^6^ School of Health Administration Faculty of Health, Dalhousie University Halifax Nova Scotia Canada; ^7^ Faculty of Science Dalhousie University Halifax Nova Scotia Canada; ^8^ Department of Clinical Nutrition, School of Public Health Iran University of Medical Sciences Tehran Iran; ^9^ Student Research Committee, School of Public Health Iran University of Medical Sciences Tehran Iran; ^10^ Clinical Tuberculosis and Epidemiology Research Center, National Research Institute of Tuberculosis and Lung Diseases (NRITLD), Masih Daneshvari Hospital Shahid Beheshti University of Medical Sciences Tehran Iran

**Keywords:** adolescents, adverse health outcomes, AMSTAR‐2, children, food insecurity, GRADE, umbrella review

## Abstract

This umbrella review examined the certainty and validity of available meta‐analyses for the association between food insecurity (FI) and adverse health outcomes (AHOs) in children and adolescents. A comprehensive systematic search was conducted using three databases: PubMed/MEDLINE, Web of Science, and Scopus, until August 20, 2024. Effect sizes were recalculated using random effects models. The GRADE tool assessed evidence certainty, while AMSTAR‐2 and the Newcastle‐Ottawa Scale evaluated study quality. Twelve meta‐analyses comprising of 108 pooled analyses (557,700 individuals) were included in this umbrella review. Food insecurity was found to be significantly associated with the increased risk of several AHOs in children and adolescents: anemia (OR: 1.50, 95% CI: 1.23 to 1.82; moderate certainty), obesity (OR: 1.16, 95% CI: 1.06 to 1.27; moderate certainty), stunting (OR: 1.12, 95% CI: 1.05 to 1.19; moderate certainty), and dental caries (OR: 1.70, 95% CI: 1.43 to 2.03; low certainty). Regarding mental health in children and adolescents, FI was found to be associated with an increased risk of suicidal ideation (OR: 1.08, 95% CI: 1.01 to 1.16; moderate certainty) and suicide plans (OR: 1.32, 95% CI: 1.14 to 1.54; moderate certainty). Further, there was found to be an increased risk in developmental outcomes (OR: 1.32, 95% CI: 1.10 to 1.59; moderate certainty) and externalizing behaviors (OR: 1.25, 95% CI: 1.08 to 1.44; low certainty). A protective association was observed for cognitive/math development disorders (OR: 0.84, 95% CI: 0.73 to 0.96; low certainty), which may be attributed to methodological limitations, though this finding requires further investigation. Food insecurity was shown to be associated with multiple AHOs in children and adolescents, with moderate to low certainty evidence. Findings should be interpreted cautiously due to substantial heterogeneity and the inclusion of low‐quality meta‐analyses. The observed heterogeneity and methodological limitations of the included meta‐analyses suggest cautious application in designing prevention programs, particularly in designing evidence‐informed nutritional and lifestyle interventions.

## Graphical abstract

This umbrella review was carried out to examine the certainty and validity of all available meta‐analyses for the association between food insecurity and adverse health outcomes in children and adolescents.

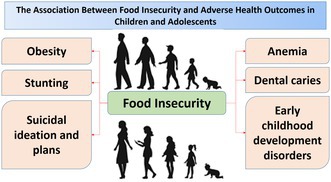

## 1 Introduction

Food insecurity (FI) has remained a major challenge in achieving global health priorities. Resulting from the limited or inadequate availability of safe, nutritious food, FI is caused by a variety of individual factors, including low income, loss of mobility, and barriers restricting transportation, access to grocery stores, and quality food (Barrett 2010). According to the FAO, approximately 828 million people were affected by undernourishment in 2021, while over 2.3 billion experienced moderate to severe FI (FAO et al. 2023). In 2024, 295 million people in 53 countries experienced severe food insecurity, according to the 2025 Global Report on Food Crises (GRFC) (Crises, Global Network Against Food 2025). Individuals experiencing FI, due to inadequate financial resources, often resort to food choices that are more cost‐effective, calorie‐dense, and readily available, resulting in less consumption of safe and nutritious food (Rosas et al. 2011).

Children and adolescents are especially susceptible to the consequences of food insecurity, which can impair both short‐term health and long‐term development, due to the nature of fast growth, changing metabolic systems, and reliance on caregivers (Council on Community Pediatrics et al. 2015). This age group also possesses increased nutritional requirements during critical periods of physical and cognitive development, especially while their psychosocial well‐being and academic performance are strongly influenced by household food availability. Consequently, FI in these formative years may disrupt developmental trajectories and increase the risk of long‐term health challenges and social disadvantages (Shankar et al. 2017; Royer et al. 2021). The dietary nutritional composition of individuals has a direct impact on their present health condition, as well as their social growth, physical and mental well‐being, and even their future health and overall welfare (Black et al. 2003). Much research conducted in the last 20 years has established a correlation between FI and certain negative developmental outcomes including impaired development of non‐cognitive abilities (i.e., interpersonal relations, self‐control), insecure attachment and less advanced mental proficiency in toddlers; higher rates of developmental risk among young children, more stomach aches, frequent headaches, and colds among children (Nord 2014, 2009). Based on the comprehensive U.S. Food Security Scale, several studies have indicated that FI is linked to lower overall health status and health‐related quality of life in children (Casey et al. 2005; Cook et al. 2004). There is evidence from a number of studies suggesting a potential link between FI and chronic conditions such as obesity, anemia, weight abnormalities, diabetes mellitus, hypertension, cancer, decline in mental health, sleep disorders, and mortality (Mazloomi et al. 2023; McLaughlin et al. 2012; Moradi et al. 2018; Moradi, Mirzababaei, Dadfarma, et al. 2019; Moradi, Mirzababaei, Mohammadi, et al. 2019; Seligman et al. 2010; Jalili et al. 2025). FI has been linked to immediate health concerns, such as undernutrition. It is also associated with longer‐term risks, including chronic conditions such as obesity, diabetes, and cardiovascular disease, which may emerge later in life (Paquin et al. 2021; Black 2012; Simonovich et al. 2020). Adverse health outcomes (AHOs) refer to harmful changes in health that can result from various causes, including medical diseases, environmental exposures, and lifestyle behaviors. These outcomes include the development of chronic diseases, exacerbation of pre‐existing health problems, and early death. Detrimental health effects may manifest structural or functional abnormalities in the body, which can reduce quality of life and lead to incapacitating illnesses (Sherwin 1983). Due to FI being linked to several detrimental health effects, it becomes a significant factor in determining health outcomes. Chronic illnesses such as obesity, diabetes, and hypertension are more common in populations that are experiencing FI (Seligman et al. 2010). There has been a noticeable increase in the number of systematic reviews and meta‐analyses (SRMAs) in recent years that have examined the relationship between FI and specific negative health‐related outcomes, such as being overweight (Zhou et al. 2023), dental caries (Sabbagh et al. 2023), stunting (Patriota et al. 2024), and health complications from under‐nutrition (Moradi, Mirzababaei, Mohammadi, et al. 2019).

Drawing generalizable conclusions is challenging as prior SRMAs varied greatly in terms of methodological rigor, populations examined, and health outcomes evaluated. The quality and consistency of these findings in children and adolescents, a group that is especially susceptible to FI‐related outcomes, have not been thoroughly assessed by any previous umbrella review. By combining and evaluating the existing meta‐analyses in this field, our study fills this gap (Aromataris et al. 2015; Pollock et al. 2017). We have conducted a comprehensive review of published meta‐analyses, evaluating the quality of evidence and the accuracy of estimates, in order to assess this association, including the risk of anemia, obesity, being overweight, being underweight, stunting, wasting, dental caries, suicidal behaviors (ideation, plan, attempt, and unspecified suicide), early childhood development disorders, externalizing behaviors, cognitive and learning‐related developmental disorders (e.g., math, reading, school readiness), motor development disorders, and hyperactivity, as well as any other outcomes for which at least one published meta‐analysis was available.

## 2 Methods

This umbrella study was conducted according to the Cochrane Handbook for the explanation of “overviews of reviews” (Higgins and Green 2011) and the “Grading of Recommendations Assessment Development and Evaluation” (GRADE) guideline (Schunemann 2008). The outcomes were also reported employing the “The Preferred Reporting Items for Overviews of Reviews” (PRIOR) guideline (Gates 2022) (Table S1). The protocol of the present study was approved with the International Prospective Register of Systematic Reviews Database (PROSPERO) (CRD42024582869).

### 2.1 Systematic Search

A search strategy (Table S2) was employed for the comprehensive systematic search in the databases of Web of Science, Scopus, and PubMed, until August 20, 2024, without any limitation for date or language. The search found related SRMAs, evaluating the relationship between FI and the risk of AHOs in children and adolescents. One investigator (SM) was responsible for the initial assessment of the publications found, while other investigators confirmed appropriate keywords and search terms. Information from gray literature sources, including reviews, conference abstracts, case reports, notes, brief surveys, letters, and reports, was obtained by a manual search of references cited in original research articles published in the specified databases. Reference lists of all entered studies have also been reviewed for any missed literature.

### 2.2 Outcomes

The risk of anemia, obesity, being overweight, stunting, being underweight, wasting, dental caries, suicidal behaviors, and having an early childhood development disorder results reported in SRMAs of observational studies evaluating the relationship with FI in children and adolescents was eligible to include in the current study.

### 2.3 Eligibility Criteria

Inclusion criteria were defined using the PICOS framework (Table 1): (1) SRMAs of observational research (case–control, cohort, or cross‐sectional) in children and adolescent individuals (< 18 years); (2) reported data on the relationship between FI and the risk of AHOs (risk of anemia, obesity, being overweight, stunting, being underweight, wasting, dental caries, suicidal behaviors, and having an early childhood development disorder); and (3) reported odds ratios (OR), relative risk (RR), or hazard ratios (HR) with 95% confidence intervals (CIs) (Table 1). When overlap occurred, we selected the SRMA with the largest number of included trials to maximize sample size and statistical power (Neuenschwander et al. 2019). However, we also cross‐checked quality ratings and confirmed that in no case did this choice result in including a lower‐quality meta‐analysis over a higher‐quality one (Neuenschwander et al. 2019). This approach was chosen to maximize comprehensiveness, though we acknowledge that quality assessment should also be considered in future reviews. The exclusion of narrative reviews and original studies was implemented to focus specifically on systematic reviews with meta‐analyses, which provide the highest level of synthesized evidence for umbrella reviews. Two researchers (KM and BE) screened the titles/abstracts and full texts to assess eligibility. Any discrepancies were addressed by discussion between the two reviewers, and when consensus could not be reached, another researcher (SM) served as arbitrator (Table 2).

**TABLE 1:** Pecos criteria for inclusion and exclusion of the studies.

Parameter	Criteria
Population	Children and adolescents (< 18 years old)
Exposure	Food insecure household
Comparator	Food secure household
Outcomes	Risk of anemia, obesity, overweight, stunning, underweight, wasting, dental caries, suicidal behaviors, and early childhood development disorders
Study design	Systematic reviews and meta‐analyses

**TABLE 2:** The association between food insecurity and children's adverse health outcomes risk.

	Number of effect size	Number of participants	Odds ratio (95% CI)	*p*	*I* ^2^ (%)	*p*_heterogeneity_	Egger's test	Certainty of evidence (GRADE)
**Adverse health outcomes**								
Anemia	19	63,996	1.50 (1.23 to 1.82)	0.000	84.7	< 0.001	0.091	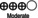
Obesity	21	111,286	1.16 (1.06 to 1.27)	0.001	50.7	0.004	0.177	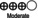
Stunting	19	57,167	1.12 (1.05 to 1.19)	0.000	80.3	< 0.001	0.195	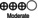
Wasting	5	2701	1.04 (0.96 to 1.13)	0.306	0.0	0.667	0.252	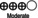
Dental caries	16	129,774	1.70 (1.43 to 2.03)	< 0.001	62.5	< 0.001	0.992	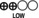
Underweight	13	37,063	1.16 (0.98 to 1.38)	0.089	75.5	< 0.001	0.342	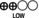
Overweight	11	50,389	1.08 (0.99 to 1.18)	0.087	62.0	0.003	0.530	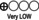
**Suicidal behaviors (adolescents)**								
Suicide ideation	30	82,869	1.08 (1.01 to 1.16)	0.033	99.7	< 0.001	0.934	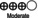
Suicide plan	11	27,667	1.32 (1.14 to 1.54)	0.000	99.1	< 0.001	0.594	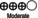
Suicide attempt	18	44,656	1.05 (0.91 to 1.22)	0.495	99.0	< 0.001	0.039	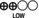
Unspecified suicide	2	7662	1.54 (0.60 to 3.98)	0.368	85.9	0.008	—	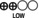
**Early childhood development disorders (children)**								
Developmental risk	3	75,144	1.32 (1.10 to 1.59)	0.003	49.1	0.140	—	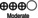
Motor	2	8204	0.91 (0.80 to 1.04)	0.175	0.0	0.688	—	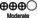
Cognitive/vocabulary	2	10,990	0.92 (0.83 to 1.02)	0.097	48.5	0.163	—	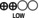
Cognitive/math	3	13,392	0.84 (0.73 to 0.96)	0.010	0.0	0.784	—	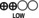
Cognitive/school readiness and reading	3	14,646	0.91 (0.82 to 1.00)	0.055	0.0	0.385	—	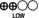
Externalizing behavior	6	14,211	1.25 (1.08 to 1.44)	0.002	74.8	0.001	0.312	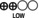
Externalizing behavior/hyperactivity	3	6738	1.18 (0.79 to 1.76)	0.418	83.3	0.002	—	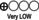
Internalizing behavior	2	3038	1.48 (0.70 to 3.15)	0.305	92.9	< 0.001	—	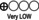
Internalizing behavior/anxiety	3	2518	1.14 (0.82 to 1.57)	0.433	92.7	< 0.001	—	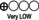

### 2.4 Data Extraction

Two researchers (SM and KM) extracted the below data from SRMAs independently: last name of the first author and publication year, average age, country, sex, effect size (OR with 95% CI), number of studies entered in the largest and most complete meta‐analyses, number of studies in comparative meta‐analyses that used similar supplementation, as well as the number of individuals in either studies. Some outcomes were represented by fewer than three meta‐analyses, which restricts the certainty of evidence and limits the feasibility of subgroup or sensitivity analyses. We also extracted additional information from every study entered in selected SRMAs: age, number of participants, countries, sex, and exposure assessment tool.

### 2.5 Assessment of Methodological Quality

Two researchers (KM and MK) evaluated the overall quality of the selected SRMAs by employing “A Measurement Tool to Assess Systematic Reviews” (AMSTAR2) (Shea et al. 2017) and included original studies evaluated by applying the Newcastle‐Ottawa Scale (NOS) tool (Wells 2000). Any discrepancies were addressed by discussion between reviewers, with arbitration by another researcher (SM) when needed.

### 2.6 Data Synthesis

Odds ratio (OR) and their 95% CIs were extracted from original studies interred in the SRMA. Considering both within‐and between‐study heterogeneity, the ORs with 95% CIs illustrated in the forest plot of every meta‐analysis were reassessed, employing a conservative random‐effects model (DerSimonian and Laird 1986). Heterogeneity was evaluated by applying the *I* ^2^ statistics while a chi‐square test was conducted for homogeneity (P‐heterogeneity > 0.10). Heterogeneity was categorized as low (< 25%), moderate (25%–50%), high (50%–75%), or very high (> 75%) following established guidelines (Cumpston et al. 2019). Furthermore, meta‐regression analyses were performed when 10 or more study arms were available (Chandler et al. 2019) and aimed to examine whether race/ethnicity, sex, age and FI assessment tools modified the relationship between FI and AHOs. Several outcomes did not meet this threshold and therefore meta‐regression could not be performed, including: wasting (5 effect sizes), motor development disorder (2 effect sizes), cognitive/vocabulary (2 effect sizes), cognitive/math (3 effect sizes), cognitive/school readiness and reading (3 effect sizes), externalizing behavior/hyperactivity (3 effect sizes), internalizing behavior (2 effect sizes), internalizing behavior/anxiety (3 effect sizes), and unspecified suicide (2 effect sizes). Meta‐regression analyses were successfully conducted only for outcomes with ≥ 10 effect sizes (anemia, obesity, stunting, dental caries, underweight, overweight, suicide ideation, suicide plan, suicide attempt, developmental risk, and externalizing behavior), as reported in Table S6.

Sensitivity analysis was conducted by omitting each study and evaluating the remaining pooled effect estimates. We conducted Egger's test to assess the likelihood of publication bias (Egger et al. 1997). All analyses were conducted by Stata version 16.0 (StataCorp). *p* < 0.05 was considered as statistical significance.

### 2.7 GRADE Rating

The GRADE methodology was applied to evaluate the quality of evidence (Guyatt et al. 2011). Each outcome obtained from intervention trials was initially regarded as high; however, it can be adjusted up or down based on pre‐determined criteria.

## 3 Results

### 3.1 Search Results

**FIGURE 1:**
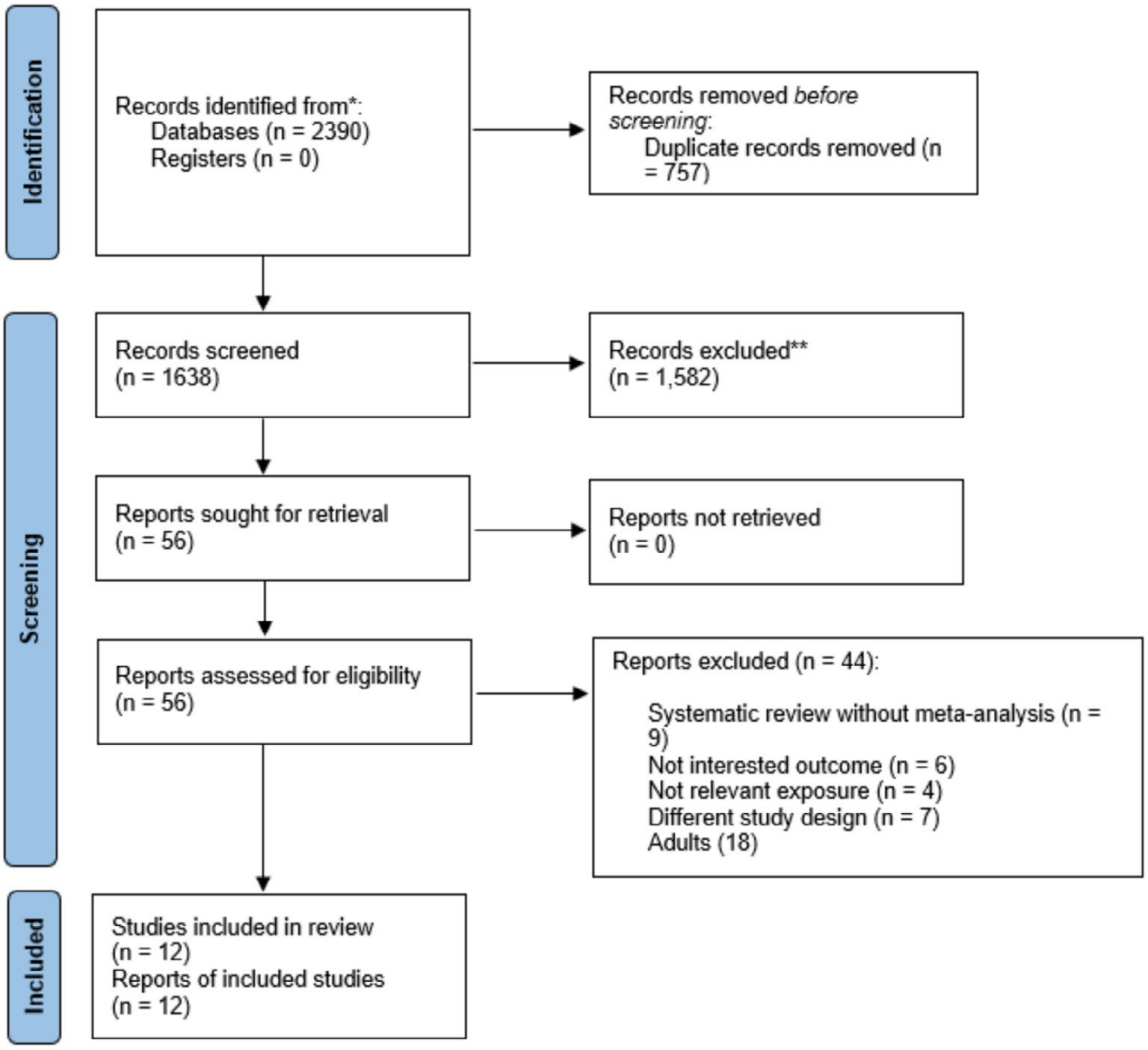
Flow diagram.

A systematic literature search identified 1638 without duplicate records (Figure 1). Next, studies were assessed for eligibility, and 44 were excluded for reasons detailed in Table S3. Finally, twelve meta‐analyses (Moradi et al. 2018; Moradi, Mirzababaei, Mohammadi, et al. 2019; Zhou et al. 2023; Sabbagh et al. 2023; Patriota et al. 2024; Belachew and Tewabe 2020; de Oliveira et al. 2020; Derakhshandeh‐Rishehri et al. 2022; Drumond et al. 2023; Eskandari et al. 2022; Kaggwa et al. 2023; Pourmotabbed et al. 2020) with 108 distinct pooled analyses were included.

### 3.2 Study Features

The AHO results in children and adolescents revealed to include anemia, obesity, being overweight, stunting, being underweight, wasting, dental caries, suicidal behaviors, and early childhood development disorders. All included studies focused on children and adolescents as the main study population. These studies were published in the last decade (between 2018 and 2024). All selected meta‐analysis articles examined the non‐dose–response association between FI and AHOs. The number of articles included from systematic reviews that were selected for pooled analysis varied from 2 to 30. Generally, 557,700 individuals were included across the pooled analyses, and study participants ranged from 50 to 225,965 (see Table S4). Among the original articles included, 87 studies (with 417,096 subjects) had cross‐sectional design, and 30 articles (with 140,604 participants) had cohort or longitudinal settings. The included original articles used several FI assessment tools including ELCSA, HFIAS, HFSSM, USFSSM, Brazilian Food Insecurity Scale, Radimer/Cornell Hunger and Food Insecurity instrument, The 7‐item Hunger Scale, 12‐month Food Security Scale, and study or local questionnaires. All included studies were conducted on children and adolescents as the main subjects.

### 3.3 Syntheses Results

All reported pooled ORs were derived from our recalculations rather than relying on the original publications. The estimated effect sizes using equivalent OR relationships between FI and the risk of children and adolescent AHOs are displayed in Table 2. The random model effect results indicated that greater FI exposure was significantly related to an increased risk of 10 (55.5%) children and adolescent AHOs.

The pooled analysis demonstrated a positive association between higher FI exposure and risk of anemia (OR: 1.50, 1.23 to 1.82; medium certainty), obesity (OR: 1.16, 1.06 to 1.28; medium certainty), stunting (OR: 1.12, 1.05 to 1.19; medium certainty), dental caries (OR: 1.70, 1.43 to 2.03; low certainty). However, there was no association between FI exposure and the risk of wasting (OR: 1.04, 0.96 to 1.13; medium certainty), being underweight (OR: 1.16, 0.98 to 1.38; low certainty), or overweight (OR: 1.08, 0.99 to 1.18; very low certainty).

Moreover, the pooled analysis indicated a direct relationship between higher FI exposure and the risk of suicidal ideation (OR: 1.08, 1.01 to 1.16; medium certainty), suicide plan (OR: 1.32, 1.14 to 1.54; medium certainty), unlike the risk of performing a suicide attempt (OR: 1.05, 0.91 to 1.22; low certainty) or unspecified suicide (OR: 1.54, 0.60 to 1.98; low certainty).

In addition, we observed evidence supporting a positive relationship between higher FI exposure and the risk of having a developmental disease (OR: 1.32, 1.10 to 1.59; moderate certainty) and externalizing behavior (OR: 1.25, 1.08 to 1.44; low certainty). Although there was a reverse relationship between higher FI exposure and the risk of having a cognitive/math developmental disorder (OR: 0.84, 0.73 to 0.96; low certainty). There was, however, shown to be no association between FI exposure and the risk of having a motor development disorder (OR: 0.91, 0.80 to 1.04; medium certainty), cognitive/school readiness and reading development disorder (OR: 0.91, 0.82 to 1.00; low certainty), or externalizing behavior/hyperactivity (OR: 1.18, 0.79 to 1.76; very low certainty).

### 3.4 Sensitivity Analyses and Publication Bias

Sensitivity analyses across the highest to the lowest meta‐analysis for AHOs showed no significant influence in any single article (Figure S1). Egger's test results for publication bias assessment showed no significant publication bias for most outcomes (*p* > 0.05). However, significant publication bias was detected regarding the risk of performing a suicide attempt (*p* = 0.039). The detailed results of Egger's test for all outcomes are presented in Table 2.

### 3.5 Meta‐Regression Analysis

Table S6 displays the results of the meta‐regression analyses. No significant impact on most AHOs risk was observed with race/ethnicity, sex, age, and FI assessment tools (*p* > 0.05). However, the results revealed that race/ethnicity (*p* = 0.004) and FI assessment tools (*p* = 0.031) modify the association between FI and anemia risk among children and adolescents.

### 3.6 Quality Assessment

The quality evaluation results for the included SRMAs are reported in Table S7. The outcomes suggested that evidence of quality are “high”, “low” and “critically low” at 33.3%, 50.1%, and 16.6%, respectively. Among the selected SRMAs, two (Zhou et al. 2023; Belachew and Tewabe 2020) of them were categorized as critically low quality. The inclusion of these critically low‐quality SRMAs may have introduced bias into our findings, and sensitivity analyses excluding these studies could provide more robust estimates. The most important limitations leading to a rating of critically low quality were related to the absence of pre‐established review methods and the lack of reporting on funding sources.

## 4 Discussion

### 4.1 Core Declaration

This umbrella review provides a comprehensive analysis and evaluation of the current evidence concerning the relationships between FI and AHOs in children and adolescents. The combined analyses revealed a clear link between FI and an increased likelihood of negative health effects, such as obesity, anemia, stunting, and dental caries. Additionally, FI was associated with suicidal behaviors among adolescents, encompassing both suicidal ideation and having a suicide plan. Being exposed to FI was also associated with developmental disorders in early childhood, encompassing developmental risks, as well as externalizing behaviors in children. However, there was a reverse relationship between higher FI exposure and the risk of having a cognitive/math developmental disorder in children. The findings of this umbrella review, however, did not reveal any significant associations between FI and the various risks linked to being underweight, overweight, and wasting. Moreover, our study's results did not find any association between FI and the risk of unspecified suicide and suicide attempts in adolescents. Further, there was no association between FI exposure and the risk of having a motor development disorder, cognitive/school readiness and reading development disorder, or externalizing behavior/hyperactivity. The possible pathways through how FI may lead to negative health outcomes are elucidated as follows.

One of the most important findings emanating from the quality assessment was that only 33.3% of included systematic reviews and meta‐analyses were determined as being high quality, 50.1% as low, and 16.6% as critically low, primarily due to their lack of pre‐specified review plans and inadequate reporting of funding sources. Inclusion of low and critically low‐quality studies poses a threat to the validity of certain conclusions. Therefore, the inclusion of such low‐quality SRMAs in an umbrella review would undermine the overall strength and coherence of evidence and render it more challenging to make solid conclusions regarding the relationship between FI and AHOs in children and adolescents in our context.

### 4.2 Food Insecurity and Adverse Health Outcomes

The variation in food quality accessible to individuals across different income brackets can be attributed to the significant expenses linked to maintaining a nutritious and well‐rounded diet. Less healthy and less nutrient‐dense diets are generally less expensive per calorie, thus more accessible to poor‐income groups (Moradi et al. 2018), while in contrast, healthier diets are consistently more expensive (Darmon and Drewnowski 2015). Children and adolescents living in food insecure households often lack sufficient access to high quality protein, essential micronutrients, and bioavailable minerals such as iron, vitamin B12, and folate (Rodríguez et al. 2017; Jun et al. 2021; Kirkpatrick and Tarasuk 2008; Eicher‐Miller et al. 2009; Qasrawi et al. 2024). However, FI is strongly associated with an increased intake of energy‐dense, sugar and fat‐rich foods (Fram et al. 2015), and in turn, it may a plausible mechanism for association between FI and increased risk of obesity. Potochnick et al. in Hispanic/Latino adolescents in the United States, found a 46% FI rate, which is twice the national average. The authors found FI to be linked with higher BMI and depression scores, which was mediated by higher economic stress, acculturative stress, and lower family support. The connection was strongest in adolescents in the lowest‐income families, suggesting that FI‐related weight gain is amplified in the context of poverty (Potochnick et al. 2019).

Our findings demonstrated a clear association between FI and stunting. Stunting has been linked with low vitamin A levels, and lack of supplementation can further lead to iron‐deficiency anemia (Dessie et al. 2024). FI has been shown to contribute to chronic undernutrition, which is the primary cause of stunting in children. Several reasons are responsible for the heightened risk of stunting among food‐insecure families. Low protein, high sugar diets, and inadequate intake of vitamins and minerals such as vitamin D and zinc are significant considerations (El Bilbeisi et al. 2022; Santin et al. 2016). FI mothers also tend to give birth to low birth weight infants (Chowdhury et al. 2018), an established risk factor for stunting (Halli et al. 2022). In addition, children experiencing FI are often in locations with poor sanitation and inadequate access to healthcare, which increases their risk for multiple reinfections, including diarrhea (Chakraborty et al. 2023). These results are problematic for food‐insecure children as they affect nutritional absorption and increase nutritional loss, leading to even more chances to develop or maintain stunting (Gorospe and Oxentenko 2012).

Additionally, the current study showed there to be an association between FI and an increased risk of anemia. FI is strongly associated with limited access to iron‐rich foods (e.g., red meat, legumes, fortified cereals) and low intake of nutrients such as vitamin C and vitamin A, which are crucial for enhancing the bioavailability of iron (Qasrawi et al. 2024; Silva et al. 2021; Mejia and Erdman 2025). Furthermore, FI in pregnancy elevates the maternal risk of anemia, consequently increasing children's risk of anemia (Singh et al. 2021). Additionally, prolonged bottle‐feeding and excessive consumption of milk during child infancy may replace iron‐rich foods and block the absorption of iron, thereby causing iron deficiency anemia (Mejia and Erdman 2025; Singh et al. 2021). Additionally, the outcome of the meta‐regression analysis showed that race/ethnicity and the FI method were significant moderators of the relationship between FI and anemia risk. Differences in dietary intake (Rawal et al. 2020; Li et al. 2025), socioeconomic inequities (Rocha et al. 2020), and access to health care (Hassan et al. 2024) by ethnic groups may partly explain why FI is associated with a stronger or weaker risk of anemia in some groups. Additionally, the use of varied FI assessment methods (e.g., HFSSM, HFIAS, ELCSA) may create differences in exposure classification, which may affect the strength of the observed associations. Our findings suggest that both context and assessment methods are an important factor in the association between FI and anemia, underscoring the need for standardized FI measurements in future studies.

FI is one of the determinants of dental caries among children due to the interactions between food behaviors, limited access to dental care, and socio‐economic stressors. FI contributes to increased dental caries through the stimulation of high consumption of sugar (Landry et al. 2019), the reduction of nutrient intake such as calcium and vitamin D (Kelters et al. 2025), and limited access to health care including dental care (Jackson and Testa 2021; Lee et al. 2023; Bahanan et al. 2021). This is further promoted by socio‐economic stress and neglectful behavior, particularly among vulnerable families.

While the majority of outcomes within this umbrella review found anticipated correlations between FI and negative health outcomes, some null correlations need further examination. Of particular interest, no significant correlation was found between FI and underweight status, notwithstanding the theoretical presumption that limitations in food access would result in lower body weight. This apparent contradiction can be attributed to adaptive strategies at the household level. Parents in food‐insecure households often prioritize feeding their children, even if it means consuming less food or less nutritionally dense foods themselves (Hevesi et al. 2024). This dynamic obscures the true relationship between FI and the prevalence of underweight conditions in children and adolescents, making it difficult to identify the underlying issues. Furthermore, FI can also result in decreased food quality, not only in terms of lower nutrient density (i.e., reduced intake of fruits, vegetables, and protein), but also in terms of food safety. It is essential that food safety is a natural aspect of food quality, and FI households may seek low quality or unsafe sources of food due to its accessibility in relation to high quality or safe food sources. In addition, the etiological pathways of these indices are different. Wasting is usually an indicator of acute malnutrition (WHO 2013), whereas FI is more likely to represent long‐term limitations in food access that lead to micronutrient deficiency and impaired growth such as stunting (de Onis and Branca 2016), without evidence of short‐term weight loss. Additionally, FI often results in the consumption of calorie‐dense, nutrient‐poor foods (Angeles‐Agdeppa et al. 2021). This effect can prolong or even increase weight without accounting for micronutrient requirements, which may influence the associations with being underweight or overweight. Additional methodological concerns, such as heterogeneity of FI assessment tools and reliance on cross‐sectional designs, could further contribute to underestimating true associations. Therefore, non‐significant findings for being underweight, overweight, and wasting should not be interpreted as an absence of effect; instead, their findings could highlight complex FI pathways and the need for longitudinal studies.

The lack of correlation between FI and wasting, acute malnutrition (Ewune et al. 2022), can be explained by the chronic nature of food insecurity. Families can adapt in the long term to chronic FI by reducing the quality of their diet (micronutrient deficiency), in place of reducing energy intake to levels that result in wasting. Additionally, wasting has a multifactorial etiology (e.g., infection, acute illness) extending beyond food availability alone.

### 4.3 Food Insecurity and Suicidal Behavior in Adolescents

Suicidal behavior is a chain of actions and ideas regarding harming oneself, such as suicidal ideation, planning, and suicide attempts (Klonsky et al. 2016; Liu and Miller 2014). The prevalence of suicidal behaviors in adolescents is significantly elevated in low‐ and middle‐income countries. In a study among 46 countries, suicidal thoughts were reported at 14.5%, planning was at 14.6%, and attempts were at 12.7% (Li et al. 2021). Additionally, the findings indicated that suicidal thoughts and planning were more common among adolescents aged 14 to 15 than in those aged 12 to 13 (Li et al. 2021). FI is a stressor that adversely affects adolescent health. FI has a strong association with mental illness such as depression and anxiety (McLaughlin et al. 2012; Shankar et al. 2017; Poole‐Di Salvo et al. 2016), and has been revealed to impair cognitive and emotional functioning, which leads to suicidal ideation and behavior (Van Orden et al. 2010). Adolescents who reside in food‐insecure homes feel shame, depression, family tension, stigma, and social isolation (Leung et al. 2020; Shtasel‐Gottlieb et al. 2015). When these affective states are viewed as being fixed, coupled with reducing fear of death, they can lead to suicidal behavior (Van Orden et al. 2010). FI is also associated with malnutrition and micronutrient deficiencies (e.g., calcium, folate, zinc) (Basiry et al. 2024). Low levels of these nutrients have been shown to be correlated with depression (Shen et al. 2023; Liwinski and Lang 2023; Rafalo et al. 2016), a proven suicide risk factor (Hallfors et al. 2004). Suicidal attempts are also frequently typified by reduced consumption of meat, fish, fruits, and vegetables in relation to non‐attempting counterparts (Li et al. 2009). However, there is no strong correlation of FI with attempted suicide or unspecified suicide in our analysis. Thus, though FI is a serious stressor, it cannot be an immediate or sole cause of suicidal behavior. The contrast in the levels of suicidal behavior observed in this research indicates that FI is associated with suicidal ideation and planning more strongly than with suicide attempts. FI is a source of chronic psychosocial stress, depression, anxiety, shame, and social isolation (Leung 2020; Park and Berkowitz 2024), all of which increase the possibility of ideation and planning. Suicide attempt is more commonly the result of multifactorial interaction involving mental illness (Park et al. 2018), substance abuse (Pompili et al. 2012), and social isolation (Motillon‐Toudic et al. 2022). FI is not directly related to these risk factors. Moreover, data might underestimate true cases due to stigma or absence of medical records. In contrast, planning and idea generation are reported more commonly on surveys and are thus more likely to be captured in research. In addition, evidence of publication bias was noted in our investigation for suicide attempt; therefore, this outcome may be influenced by incomplete representation of the evidence base in available studies. Bias can help to explain the lack of a statistically significant association found in our pooled analysis, as null or negative findings may have been underreported and distorted the pooled estimate. Further research with more rigorous and comprehensive reporting needs to be conducted to clarify this association.

### 4.4 Food Insecurity and Early Childhood Development Disorders

Children are particularly vulnerable to the long‐term consequence of FI, resulting in undernutrition and micronutrient deficiency, with negative consequences on growth and development (Kirkpatrick and Tarasuk 2008). As the current study revealed, FI was related with developmental risks, as well as externalizing behaviors in children. Food insecurity is associated with a decline in the quality of dietary intake (Kohanmoo et al. 2024), resulting in nutrient deficiencies among young children (Jun et al. 2021). This situation results in malnutrition that can subsequently elevate the risk of developmental problems in children, adversely affecting brain development (Sethi et al. 2022) and contributing to behavioral problems (Liu and Raine 2011). FI also disrupts mealtime routines as, with inconsistent access to healthy foods, it becomes increasingly difficult for parents to provide regular meals (Schuler et al. 2020). Notably, the result of the current study revealed that there was a reverse relationship between higher FI exposure and the risk of having a cognitive development disorder. This finding can be explained by FI families experiencing lower access to diagnostic services, and hence under‐diagnosis or late diagnosis of cognitive disorders among children may be likely. Parent‐report data may also lead to underreporting due to stigma or prioritization of health issues that immediately endanger the life of their children. Therefore, the noted inverse relationship between FI and cognitive/math developmental disorders should be interpreted carefully due to this association being largely a result of methodological concerns, including under‐diagnostic practices, the reliance on parent‐reported measures, and the accessibility of diagnostic services in food insecure homes. Future studies using longitudinal designs, standardized developmental assessment measures, and realistic assessments with greater detail for diagnostic purposes, and collective investigations, would help address unanswered questions. It is important for future investigation to help clarify if the finding of FI and cognitive/math developmental disorders capture a protective effect or are merely measurement and reporting biases.

In addition, our study found an association between FI and externalizing behaviors in children; however, not with hyperactivity. This finding may be explained by the nature of externalizing behaviors, their symptoms being described as aggression, delinquency, and hyperactivity, with each having a varying effect that could be attributed to or exacerbated by FI. In example, FI would likely affect aggression, a common stress response in children who live in food insecure conditions (Jackson et al. 2018).

This study did not find any significant relationship between FI and challenges in children's motor development. Explanations for this finding are most likely attributed to the greater set of genetic and environmental determinants that are stronger in their influence on motor skill development (Zi et al. 2023; Venetsanou and Kambas 2010) than the prevalence of food security.

### 4.5 Limitations and Strengths

This umbrella review possesses numerous strengths, notably in its systematic approach to searching, gathering, and evaluating the credibility of evidence obtained from a variety of systematic reviews. It incorporates data from meta‐analyses of epidemiological studies that examine the connections between FI and AHOs in children and adolescents, encompassing a diverse array of participants and research. Furthermore, our review meticulously assessed the methodological quality of the included meta‐analyses. This comprehensive analysis offers an in‐depth understanding of the implications of FI by addressing a broad spectrum of health consequences in children and adolescents. However, it is crucial to acknowledge the numerous potential limitations inherent in the present umbrella review, which should be considered when analyzing our findings. The research conducted employed various instruments to evaluate food insecurity. While the diverse metrics utilized in these studies possess both validity and reliability, the lack of comparability among these measures represents a significant gap that warrants attention. Additionally, the observational design of the studies incorporated implies that, although it is possible to recognize associations, establishing a definitive causal relationship remains unattainable. Furthermore, although most of the primary studies incorporated in this review accounted for significant confounding variables, it is crucial to recognize that residual confounding could remain. Finally, we acknowledge that the observed high heterogeneity in some analyses likely reflects mentioned differences in study populations, food insecurity assessment tools, and geographical regions across included studies. Despite these limitations, we have taken several measures to ensure the quality of our findings. First, we included studies that employed validated and reliable measures of assessing food insecurity, enhancing the consistency of the evidence despite heterogeneity of measurement tools. In addition, by combining a large number of studies with heterogeneous populations and settings, this review enhances the external validity of the results. In addition, we utilized the AMSTAR 2 tool to assess the quality of each study included to differentiate between higher and lower quality evidence. We evaluated the certainty of the evidence in accordance with the GRADE criteria, ensuring transparent and interpretable findings. Collectively, these approaches helped to minimize bias and increase the credibility and generalizability of our findings for policy and clinical practice.

### 4.6 Application in Policymaking

In order to enhance food security and improve the quality of diets among children and adolescent populations, it is essential to implement policy modifications within school programs that focus on nutrient‐rich food systems available in educational institutions (Beresford et al. 2001). Lowering the price of nutritious meals and snacks leads to an increase in high‐quality, nutrient‐dense food consumption among consumers, including families and children (French et al. 2001). In addition to general improvements to diet, policy actions should also aim to remedy specific deficiencies due to malnutrition identified in this umbrella review. For example, the increase in the availability of iron‐rich foods in school feeding programs, such as fortified cereals, could help address the increased risk of anemia from FI. Similarly, strategies to increase access to foods rich in calcium and vitamin D could address the increased risk of dental caries. It is essential to focus on improving both the accessibility and adequacy of various nutritional assistance initiatives (Hartline‐Grafton and Hassink 2021). In addition, it should be noted that although our study concentrated mainly on food quality, food safety is an integral part of food quality in general (Sikora 2005). It is imperative that foods be free of contaminants, pathogens, and toxicants to allow the desired health advantages of any dietary regimen to be brought into effect. Policies to improve public health through nutrition should therefore incorporate food safety standards and protocols, especially in facilities where exposure to unsafe food can undermine nutritional goals.

## 5 Conclusion

This umbrella review finds FI to be linked to an increased likelihood of various AHOs in children and adolescents, such as anemia, obesity, stunting, dental caries, suicidal ideation and plans, developmental risks, as well as cognitive/math, and externalizing behaviors. The results of this study underscore the necessity for robust public health initiatives aimed at addressing FI and its adverse impact on the health and development of children. It is imperative that subsequent research delves deeper into the mechanisms that connect these variables and assesses the efficacy of interventions intended to alleviate FI and enhance health outcomes for this at‐risk demographic.

## Author Contributions

**Seyedeh Parisa Moosavian:** funding acquisition (equal), resources (equal). **Farhang Hameed Awlqadr:** supervision (equal), writing – original draft (equal). **Sanaz Mehrabani:** writing – original draft (equal). **Kimia Mazinani:** funding acquisition (equal), visualization (equal). **Faramarz Jalili:** data curation (equal), validation (equal). **Felicity MacIsaac:** writing – review and editing (equal). **Seyed Mojtaba Ghoreishy:** writing – original draft (equal). **Behzad Ebrahimi:** formal analysis (equal), visualization (equal). **Mohammad Ali Hojjati Kermani:** funding acquisition (equal), methodology (equal). **Sajjad Moradi:** conceptualization (equal), data curation (equal), formal analysis (equal), funding acquisition (equal), investigation (equal), methodology (equal), project administration (equal), resources (equal), software (equal), supervision (equal), validation (equal), visualization (equal), writing – original draft (equal), writing – review and editing (equal).

## Funding

The authors have nothing to report.

## Ethics Statement

The authors have nothing to report.

## Conflicts of Interest

The authors declare no conflicts of interest.

## Supporting information


**Appendix S1:** fsn371625‐sup‐0001‐Supinfo.docx.

## Data Availability

The datasets generated and/or analyzed during the current study are available from the corresponding author on reasonable request.
