# Supplementary material for: The Association Between Food Insecurity and Adverse Health Outcomes in Children and Adolescents: An Umbrella Review
Source: Food Sci Nutr. 2026 Apr 12;14(4):e71625. doi: 10.1002/fsn3.71625 (PMC13071150; doi:10.1002/fsn3.71625)
Supplement: Supplementary file 1 — Appendix S1: fsn371625‐sup‐0001‐Supinfo.docx. [file FSN3-14-e71625-s001.docx]

| **Content** | **Page** |
| --- | --- |
| Supplementary Table 1 | 2-4 |
| Supplementary Table 2 | 5 |
| Supplementary Table 3 | 6-8 |
| Supplementary Table 4 | 9-16 |
| Supplementary Table 5 | 17 |
| Supplementary Table 6 | 18 |
| Supplementary Table 1 | 19 |
| Supplementary Figure 1 | 20-31 |
| Supplementary references | 32-38 |

**Supplementary Table 1.** PRIOR checklist.

| **Section**  Topic | | **#** | | **Item** | | **Location reported** | |
| --- | --- | --- | --- | --- | --- | --- | --- |
| **TITLE** | | | | | |  | |
| Title | | 1 | | Identify the report as an overview of reviews. | | Page 1 | |
| **ABSTRACT** | | | | | |  | |
| Abstract | | 2 | | Provide a comprehensive and accurate summary of the purpose, methods, and results of the overview of reviews. | | Page 2 | |
| **INTRODUCTION** | | | | | |  | |
| Rationale | | 3 | | Describe the rationale for conducting the overview of reviews in the context of existing knowledge. | | Page 4 | |
| Objectives | | 4 | | Provide an explicit statement of the objective(s) or question(s) addressed by the overview of reviews. | | Page 5 | |
| **METHODS** | | | | | |  | |
| Eligibility criteria | | 5a | | Specify the inclusion and exclusion criteria for the overview of reviews. If supplemental primary studies were included, this should be stated, with a rationale. | | Page 6 | |
|  |  | 5b | | Specify the definition of ‘systematic review’ as used in the inclusion criteria for the overview of reviews. | | Page 6 | |
| Information sources | | 6 | | Specify all databases, registers, websites, organizations, reference lists, and other sources searched or consulted to identify systematic reviews and supplemental primary studies (if included).  Specify the date when each source was last searched or consulted. | | Page 5 | |
| Search strategy | | 7 | | Present the full search strategies for all databases, registers and websites, such that they could be reproduced. Describe any search filters and limits applied. | | Table S2 | |
| Selection process | | 8a | | Describe the methods used to decide whether a systematic review or supplemental primary study (if included) met the inclusion criteria of the overview of reviews. | | Page 5 | |
|  |  | 8b | | Describe how overlap in the populations, interventions, comparators, and/or outcomes of systematic reviews was identified and managed during study selection. | | Table 1 | |
| Data collection process | | 9a | | Describe the methods used to collect data from reports. | | Page 6 | |
|  |  | 9b | | If applicable, describe the methods used to identify and manage primary study overlap at the level  of the comparison and outcome during data collection. For each outcome, specify the method used to illustrate and/or quantify the degree of primary study overlap across systematic reviews. | | Page 7 | |
|  |  | 9c | | If applicable, specify the methods used to manage discrepant data across systematic reviews during data collection. | | Page 6 | |
| Data items | | 10 | | List and define all variables and outcomes for which data were sought. Describe any assumptions made and/or measures taken to identify and clarify missing or unclear information. | | Page 6 | |
| Risk of bias assessment | | 11a | | Describe the methods used to *assess* risk of bias or methodological quality of the included systematic reviews. | | Page 7 | |
|  |  | 11b | | Describe the methods used to *collect* data on (from the systematic reviews) and/or *assess* the risk of bias of the primary studies included in the systematic reviews. Provide a justification for instances where flawed, incomplete, or missing assessments are identified but not re-assessed. | | Page 7 | |
|  |  | 11c | | Describe the methods used to *assess* the risk of bias of supplemental primary studies (if included). | | Page 7 | |
| Synthesis methods | | 12a | | Describe the methods used to summarize or synthesize results and provide a rationale for the choice(s). | | Page 7 | |
|  |  | 12b | | Describe any methods used to explore possible causes of heterogeneity among results. | | Page 8 | |
|  |  | 12c | | Describe any sensitivity analyses conducted to assess the robustness of the synthesized results. | | Not applicable | |
| Reporting bias assessment | | 13 | | Describe the methods used to *collect* data on (from the systematic reviews) and/or *assess* the risk of bias due to missing results in a summary or synthesis (arising from reporting biases at the levels of the systematic reviews, primary studies, and supplemental primary studies, if included). | | Page 7 | |
| Certainty assessment | | 14 | | Describe the methods used to *collect* data on (from the systematic reviews) and/or *assess* certainty (or confidence) in the body of evidence for an outcome. | | Page 7 | |
| **RESULTS** | | | | | |  | |
| Systematic review and supplemental primary study selection | | 15a | | Describe the results of the search and selection process, including the number of records screened, assessed for eligibility, and included in the overview of reviews, ideally with a flow diagram. | | Page 8 | |
|  |  | 15b | | Provide a list of studies that might appear to meet the inclusion criteria, but were excluded, with the main reason for exclusion. | | Table S3 | |
| **Section**  Topic | | **#** | | **Item** | | **Location reported** | |
| Characteristics of systematic reviews and supplemental primary studies | | 16 | | Cite each included systematic review and supplemental primary study (if included) and present its characteristics. | | Page 8 | |
| Primary study overlap | | 17 | | Describe the extent of primary study overlap across the included systematic reviews. | | Page 8 | |
| Risk of bias in systematic reviews, primary studies, and  supplemental primary studies | | 18a | | Present assessments of risk of bias or methodological quality for each included systematic review. | | Table S4 | |
|  |  | 18b | | Present assessments (*collected* from systematic reviews or *assessed* anew) of the risk of bias of the primary studies included in the systematic reviews. | | Table S4 | |
|  |  | 18c | | Present assessments of the risk of bias of supplemental primary studies (if included). | | Table S4 | |
| Summary or synthesis of results | | 19a | | For all outcomes, summarize the evidence from the systematic reviews and supplemental primary studies (if included). If meta-analyses were done, present for each the summary estimate and its precision and measures of statistical heterogeneity. If comparing groups, describe the direction of the effect. | | Pages 8-11 | |
|  |  | 19b | | If meta-analyses were done, present results of all investigations of possible causes of heterogeneity. | | Pages 8-11 | |
|  |  | 19c | | If meta-analyses were done, present results of all sensitivity analyses conducted to assess the robustness of synthesized results. | | Not applicable | |
| Reporting biases | | 20 | | Present assessments (*collected* from systematic reviews and/or *assessed* anew) of the risk of bias due to missing primary studies, analyses, or results in a summary or synthesis (arising from reporting biases at the levels of the systematic reviews, primary studies, and supplemental primary  studies, if included) for each summary or synthesis assessed. | | Table S4 | |
| Certainty of evidence | | 21 | | Present assessments (*collected* or *assessed* anew) of certainty (or confidence) in the body of evidence for each outcome. | | Pages 11-12 | |
| **DISCUSSION** | | | | | |  | |
| Discussion | | 22a | | Summarize the main findings, including any discrepancies in findings across the included systematic reviews and supplemental primary studies (if included). | | Page 12 | |
|  |  | 22b | | Provide a general interpretation of the results in the context of other evidence. | | Pages 12-15 | |
|  |  | 22c | | Discuss any limitations of the evidence from systematic reviews, their primary studies, and supplemental primary studies (if included) included in the overview of reviews. Discuss any limitations of the overview of reviews methods used. | | Page 15 | |
|  |  | 22d | | Discuss implications for practice, policy, and future research (both systematic reviews and primary research). Consider the relevance of the findings to the end users of the overview of reviews, e.g., healthcare providers, policymakers, patients, among others. | | Pages 15-16 | |
| **OTHER INFORMATION** | | | | | |  | |
| Registration and protocol | | 23a | | Provide registration information for the overview of reviews, including register name and registration number, or state that the overview of reviews was not registered. | | Page 5 | |
|  |  | 23b | | Indicate where the overview of reviews protocol can be accessed, or state that a protocol was not prepared. | | Page 5 | |
|  |  | 23c | | Describe and explain any amendments to information provided at registration or in the protocol. Indicate the stage of the overview of reviews at which amendments were made. | | Page 5 | |
| Support | | 24 | | Describe sources of financial or non-financial support for the overview of reviews, and the role of the funders or sponsors in the overview of reviews. | | Page 16 | |
| Competing interests | | 25 | | Declare any competing interests of the overview of reviews' authors. | | Page 16 | |
| Author information | | 26a | | Provide contact information for the corresponding author. | | Page 1 (title page) | |
|  |  | 26b | | Describe the contributions of individual authors and identify the guarantor of the overview of reviews. | | Page 16 | |
| Availability of data and other materials | | 27 | | Report which of the following are available, where they can be found, and under which conditions they may be accessed: template data collection forms; data collected from included systematic reviews and supplemental primary studies; analytic code; any other materials used in the overview of reviews. | | Page 16 | |

| **Supplementary Table 2.** Search strategy including the key terms and the queries for databases (August 20, 2024). | |
| --- | --- |
| PubMed (n=774) | #1 : ("Food Insecurity"[MeSH Terms] OR "Food Insecurity"[Title/Abstract] OR "Food Insecurities"[Title/Abstract] OR "Food Rationing"[Title/Abstract] OR "Food Supply"[MeSH Terms] OR "Food Supply"[Title/Abstract] OR "Food Supplies"[Title/Abstract] OR "Food Insecurity"[Title/Abstract] OR "Food Insecurities"[Title/Abstract] OR "Food Security"[Title/Abstract])  #2: ("Meta-Analysis"[Title/Abstract] OR "meta-analyses"[Title/Abstract] OR "Meta-Analysis"[Title/Abstract] OR "meta-analyze"[Title/Abstract] OR "Systematic Review"[Title/Abstract] OR "Systematic Reviews as Topic"[MeSH Terms] OR "Meta-Analysis as Topic"[MeSH Terms])  #3: 1 AND 2 |
| Web of Sciences  (n= 317) | #1: (TS=("food insecurity" OR "Food Insecurities" OR "Food Rationing" OR "Food Supply" OR "Food Supplies")  #2 (TS=("Meta-Analysis" OR "meta-analyses" OR "meta-analyze" OR "Systematic Review" OR "Systematic Reviews")  #3: #1 AND #2 |
| Scopus (n=1304) | #1: ( TITLE-ABS-KEY ( "Meta-Analysis" ) OR TITLE-ABS-KEY ( "meta-analyses" ) OR TITLE-ABS-KEY ( "meta-analyze" ) OR TITLE-ABS-KEY ( "Systematic Review" ) OR TITLE-ABS-KEY ( "Systematic Reviews" )  #2: TITLE-ABS-KEY ( "food insecurity" ) OR TITLE-ABS-KEY ( "Food Insecurities" ) OR TITLE-ABS-KEY ( "Food Rationing" ) OR TITLE-ABS-KEY ( "Food Supply" ) OR TITLE-ABS-KEY ( "Food Supplies" )  #3: AND #1 AND #2 |

**Supplementary Table 3.** Excluded studies with reason (n=44).

| 1. Lee, S.D., et al., *Food insecurity among postsecondary students in developed countries: A narrative review.* British Food Journal, 2018. **120**(11): p. 2660-2680. 2. Michalis, A. and V. Costarelli, *Food security research in selected Southern European and Eastern Mediterranean countries: a narrative review.* Nutrition and Food Science, 2020. **51**(4): p. 690-703. 3. Silva, E. and D. Grigsby-Toussaint, *Food insecurity in low-and middle-income countries: A narrative review.* Obesity, 2021. **29**(SUPPL 2): p. 104. 4. Aguiar, A.P. and R.D.S. Corrêa, *Intimate partner violence against woman and food insecurity: a narrative review of the literature.* Interface: Communication, Health, Education, 2022. **26**. 5. Adebiyi, V., E. Frongillo, and L. Larson, *Determinants and Consequences of Food Insecurity Among Women in Sub-Saharan Africa: A Narrative Review.* Current Developments in Nutrition, 2023. **7**. 6. McKay, F.H. and R. Bennett, *Examining the Relationship Between Food Insecurity and Family Violence: a Systematic Narrative Review.* Journal of Family Violence, 2023. 7. Varela, E.G., et al., *The Determinants of Food Insecurity Among Hispanic/Latinx Households With Young Children: A Narrative Review.* Advances in Nutrition, 2023. **14**(1): p. 190-210. | Different study design |
| --- | --- |
| 1. Askari, E., et al., *Food Insecurity among Iranian Pregnant Women: A Systematic Review and Meta-Analysis.* Iranian Journal of Nursing and Midwifery Research, 2024. **29**(4): p. 403-410. 2. Oronce, C.I.A., et al. *Interventions to address food insecurity among adults in Canada and the US: a systematic review and meta-analysis*. in *JAMA Health Forum*. 2021. American Medical Association. 3. Nkambule, S.J., et al., *Association between food insecurity and key metabolic risk factors for diet-sensitive non-communicable diseases in sub-Saharan Africa: a systematic review and meta-analysis.* Scientific reports, 2021. **11**(1): p. 5178. 4. Lopes, S.O., et al., *Food insecurity and micronutrient deficiency in adults: a systematic review and meta-analysis.* Nutrients, 2023. **15**(5): p. 1074. 5. Jung, N.M., et al., *Gender differences in the prevalence of household food insecurity: a systematic review and meta-analysis.* Public Health Nutrition, 2017. **20**(5): p. 902-916. 6. Azevedo, F.M., et al., *Food insecurity and its socioeconomic and health determinants in pregnant women and mothers of children under 2 years of age, during the COVID-19 pandemic: A systematic review and meta-analysis.* Frontiers in Public Health, 2023. **11**: p. 1087955. | Not interested outcome |
| 1. Jung, N.M., et al., *Gender differences in the prevalence of household food insecurity: a systematic review and meta-analysis.* Public Health Nutrition, 2017. **20**(5): p. 902-916. 2. Boneya, D.J., A.A. Ahmed, and A.W. Yalew, *The effect of gender on food insecurity among HIV-infected people receiving anti-retroviral therapy: a systematic review and meta-analysis.* PloS one, 2019. **14**(1): p. e0209903. 3. Smith, J., et al., *Food insecurity in adults with severe mental illness: A systematic review with meta-analysis.* Journal of Psychiatric and Mental Health Nursing, 2024. **31**(2): p. 133-151. 4. Teasdale, S.B., et al., *Prevalence of food insecurity in people with major depression, bipolar disorder, and schizophrenia and related psychoses: A systematic review and meta-analysis.* Critical reviews in food science and nutrition, 2023. **63**(20): p. 4485-4502. | Not relevant exposure |
| 1. Laurentino, J.S.L., et al., *Association between food insecurity and chronic noncommunicable diseases in Brazil: a systematic review.* Rev Bras Epidemiol, 2024. **27**: p. e240041. 2. Frank, M., et al., *Association of Food Insecurity With Multiple Forms of Interpersonal and Self-Directed Violence: A Systematic Review.* Trauma, Violence, and Abuse, 2024. **25**(1): p. 828-845. 3. Williams, A., et al., *The COVID-19 pandemic and food insecurity in households with children: A systematic review.* PLoS ONE, 2024. **19**(8). 4. Magny-Normilus, C., et al., *Food insecurity and diabetes management among adults of African descent: A systematic review.* Diabetic Medicine, 2024. 5. Osei-Owusu, C., S. Dhillon, and I. Luginaah, *The impact of food insecurity on mental health among older adults residing in low- and middle-income countries: A systematic review.* PLoS ONE, 2024. **19**(3 March): p. 1-22. 6. Briggs, R., et al., *The lived experience of food insecurity among adults with obesity: a quantitative and qualitative systematic review.* Journal of Public Health (United Kingdom), 2024. **46**(2): p. 230-249. 7. Valenzuela-Guzmán, D.M. and J. Esparza-Romero, *Relationship between food insecurity and malnutrition in schoolchildren from low- and middle-income countries: a systematic review.* Nutr Hosp, 2024. 8. Whitehouse, C.R., et al., *A Systematic Review of Interventions That Address Food Insecurity for Persons With Prediabetes or Diabetes Using the RE-AIM Framework.* Science of Diabetes Self-Management and Care, 2024. **50**(2): p. 141-166. 9. Cope, A.L. and I.G. Chestnutt, *A systematic review of the association between food insecurity and behaviours related to caries development in adults and children in high-income countries.* Community Dentistry and Oral Epidemiology, 2024. | Systematic review without meta-analysis |
| 1. Abdurahman, A.A., et al., *The association of household food insecurity with the risk of type 2 diabetes mellitus in adults: a systematic review and meta-analysis.* Eur J Nutr, 2019. **58**(4): p. 1341-1350. 2. Abene, J.A., et al., *Food insecurity and binge eating: A systematic review and meta-analysis.* Int J Eat Disord, 2023. **56**(7): p. 1301-1322. 3. Aibibula, W., et al., *Food insecurity and low CD4 count among HIV-infected people: a systematic review and meta-analysis.* AIDS Care, 2016. **28**(12): p. 1577-1585. 4. Aibibula, W., et al., *Association Between Food Insecurity and HIV Viral Suppression: A Systematic Review and Meta-Analysis.* AIDS Behav, 2017. **21**(3): p. 754-765. 5. Arenas, D.J., et al., *A Systematic Review and Meta-Analysis of Food Insecurity and Dyslipidemia.* The Journal of the American Board of Family Medicine, 2022. **35**(4): p. 656-667. 6. Arenas, D.J., et al., *A Systematic Review and Meta-analysis of Depression, Anxiety, and Sleep Disorders in US Adults with Food Insecurity.* J Gen Intern Med, 2019. **34**(12): p. 2874-2882. 7. Arzhang, P., et al., *The association between food insecurity and gestational weight gain: A systematic review and meta-analysis.* Appetite, 2022. **176**: p. 106124. 8. Ayano, G., L. Tsegay, and M. Solomon, *Food insecurity and the risk of depression in people living with HIV/AIDS: a systematic review and meta-analysis.* AIDS Res Ther, 2020. **17**(1): p. 36. 9. Beltran, S., et al., *Food insecurity, type 2 diabetes, and hyperglycaemia: A systematic review and meta-analysis.* Endocrinol Diabetes Metab, 2022. **5**(1): p. e00315. 10. Beltran, S., et al., *Food insecurity and hypertension: A systematic review and meta-analysis.* PLoS One, 2020. **15**(11): p. e0241628. 11. Demétrio, F., et al., *Food insecurity in pregnant women is associated with social determinants and nutritional outcomes: a systematic review and meta-analysis.* Cien Saude Colet, 2020. **25**(7): p. 2663-2676. 12. Hidru, H.D., et al., *Prevalence of Food Insecurity and Its Associated Factors among Adult People with Human Immunodeficiency Virus in Ethiopia: A Systematic Review and Meta-Analysis.* Int J Food Sci, 2021. **2021**: p. 7816872. 13. Kantilafti, M., K. Giannakou, and S. Chrysostomou, *Multimorbidity and food insecurity in adults: A systematic review and meta-analysis.* PLoS One, 2023. **18**(7): p. e0288063. 14. Mazloomi, S.N., et al., *Food insecurity is associated with the sleep quality and quantity in adults: a systematic review and meta-analysis.* Public Health Nutr, 2022. **26**(4): p. 1-11. 15. Moradi, S., et al., *Food insecurity and adult weight abnormality risk: a systematic review and meta-analysis.* Eur J Nutr, 2019. **58**(1): p. 45-61. 16. Nguyen, G., et al., *Food insecurity during pregnancy in high-income countries, and maternal weight and diet: A systematic review and meta-analysis.* Obesity Reviews, 2024. **25**(7). 17. Pourmotabbed, A., et al., *Food insecurity and mental health: a systematic review and meta-analysis.* Public Health Nutr, 2020. **23**(10): p. 1778-1790. 18. Maddahi, M., et al., *Food Insecurity regarding the Diabetes Mellitus in Iran: A Systematic Review and Meta-Analysis.* Journal of Nutrition and Food Security, 2024. **9**(2): p. 372-382. | Adults |

| **Supplementary Table 4. Included original studies** | | | | | | | |
| --- | --- | --- | --- | --- | --- | --- | --- |
| **Outcomes** | **References** | **Author** | **Years** | **Study design** | **Population** | **Exposure measurements** | **Quality score** |
| Anemia | [1-18] | Alemayehu et al. | 2018 | Cross-sectional | 990 | HFIAS | +7/9 |
|  |  | Campbell et al. | 2011 | Cross-sectional | 4940 | HFSSM | +8/9 |
|  |  | Eicher-Miller et al. | 2009 | Cross-sectional | 11247 | HFSSM | +8/9 |
|  |  | Fischer et al. | 2014 | Cross-sectional | 5739 | ELCSA | +8/9 |
|  |  | Gubert et al. | 2016 | Cross-sectional | 4064 | Brazilian Food Insecurity Scale | +8/9 |
|  |  | Habib et al. | 2016 | Cross-sectional | 7138 | HFIAS | +8/9 |
|  |  | Jones et al. | 2016 | Cross-sectional | 4148 | ELCSA | +8/9 |
|  |  | Lisbôa et al. | 2015 | Cross-sectional | 725 | Brazilian Food Insecurity Scale | +8/9 |
|  |  | Melako et al. | 2018 | Cross-sectional | 485 | NR | +7/9 |
|  |  | Metallinos-Katsaras et al. | 2016 | Cohort | 17831 | HFSSM | +8/9 |
|  |  | Nair et al. | 2016 | Cross-sectional | 792 | HFIAS | +7/9 |
|  |  | Osei et al. | 2010 | Cross-sectional | 368 | HFIAS | +6/9 |
|  |  | Park et al. | 2009 | Cross-sectional | 2853 | HFSSM | +8/9 |
|  |  | Pirkle et al. | 2014 | Cross-sectional | 292 | HFSSM | +6/9 |
|  |  | Shen et al. | 2015 | Cross-sectional | 1583 | HFSSM | +7/9 |
|  |  | Skalicky et al. | 2006 | Cross-sectional | 626 | HFSSM | +8/9 |
|  |  | Tiku et al. | 2018 | Cross-sectional | 404 | NR | +7/9 |
|  |  | Schmeer et al. | 2017 | Cross-sectional | 431 | ELCSA | +7/9 |
| Obesity | [7, 19-33] | Jones et al. | 2016 | Cross-sectional | 4148 | ELCSA | +8/9 |
|  |  | Basirat et al. | 2012 | Cross-sectional | 310 | Radimer/Cornell Hunger and Food Insecurity instrument | +6/9 |
|  |  | Benjamin-Neelon et al. | 2020 | Cohort | 666 | HFSSM | +8/9 |
|  |  | Do et al. | 2021 | Cross-sectional | 1544 | Two-item screener. | +7/9 |
|  |  | Drennen et al. | 2019 | Cross-sectional | 28184 | HFSSM | +8/9 |
|  |  | Farzaneh et al. | 2017 | Cross-sectional | 480 | HFSSM | +7/9 |
|  |  | Gundersen et al. | 2009 | Cross-sectional | 2516 | HFSSM | +8/9 |
|  |  | Gundersen et al. | 2008 | Cohort | 1031 | Food insecurity was assessed using a three questions adapted from the HFFSM. | +8/9 |
|  |  | Holben et al. | 2015 | Cross-sectional | 7435 | HFSSM | +8/9 |
|  |  | Jafari et al. | 2017 | Cross-sectional | 365 | Radimer/Cornell Hunger and Food Insecurity instrument | +7/9 |
|  |  | Bhawra et al. | 2017 | Cross-sectional | 6900 | Local survey | +7/9 |
|  |  | Kaur et al. | 2015 | Cross-sectional | 9701 | HFSSM | +8/9 |
|  |  | Martin et al. | 2007 | Cohort | 212 | HFSSM | +6/9 |
|  |  | Metallinos et al. | 2012 | Cross-sectional | 28353 | Food insecurity was assessed using a four questions adapted from the HFFSM. | +8/9 |
|  |  | Rose et al. | 2006 | Cohort | 16889 | HFSSM | +8/9 |
|  |  | Whitaker et al. | 2006 | Cross-sectional | 2459 | HFSSM | +8/9 |
| Overweight | [5, 20, 21, 25, 26, 30, 32, 34-37] | Gubert et al. | 2016 | Cross-sectional | 4064 | Brazilian Food Insecurity Scale | +7/9 |
|  |  | Benjamin-Neelon et al. | 2020 | Cohort | 666 | HFSSM | +7/9 |
|  |  | Do et al. | 2021 | Cross-sectional | 1544 | two-item screener. | +8/9 |
|  |  | Gundersen et al. | 2008 | Cohort | 1031 | Food insecurity was assessed using a three questions adapted from the HFFSM. | +8/9 |
|  |  | Holben et al. | 2015 | Cross-sectional | 7435 | HFSSM | +8/9 |
|  |  | Martin et al. | 2007 | Cohort | 212 | HFSSM | +7/9 |
|  |  | Rose et al. | 2006 | Cohort | 16889 | HFSSM | +9/9 |
|  |  | Alaimo et al. | 2001 | Cross-sectional | 9196 | NR | +8/9 |
|  |  | Casey et al. | 2006 | Cross-sectional | 6995 | HFSSM | +8/9 |
|  |  | Dubois et al. | 2006 | Cohort | 1549 | Food insecurity was assessed using a single question. | +8/9 |
|  |  | Swindle et al. | 2018 | Cross-sectional | 808 | HFSSM | +8/9 |
| Underweight | [12, 19, 23, 30, 31, 38-45] | Osei et al. | 2010 | Cross-sectional | 368 | HFIAS | +8/9 |
|  |  | Basirat et al. | 2012 | Cross-sectional | 310 | Radimer/Cornell Hunger and Food Insecurity instrument | +7/9 |
|  |  | Farzaneh et al. | 2017 | Cross-sectional | 480 | HFSSM | +7/9 |
|  |  | Martin et al. | 2007 | Cohort | 212 | HFSSM | +7/9 |
|  |  | Metallinos et al. | 2012 | Cross-sectional | 28353 | Food insecurity was assessed using a four questions adapted from the HFFSM. | +8/9 |
|  |  | Ahmadihoseini et al. | 2019 | Cross-sectional | 240 | HFSSM | +6/9 |
|  |  | Betebo et al. | 2017 | Cross-sectional | 508 | HFIAS | +7/9 |
|  |  | Isanaka et al. | 2007 | Cross-sectional | 2526 | HFSSM | +8/9 |
|  |  | Naser et al. | 2014 | Cross-sectional | 233 | Radimer/Cornell Hunger and Food Insecurity instrument | +6/9 |
|  |  | Shahraki et al. | 2016 | Cross-sectional | 610 | HFSSM | +7/9 |
|  |  | Singh et al. | 2014 | Cross-sectional | 235 | HFIAS | +6/9 |
|  |  | Speirs et al. | 2016 | Cross-sectional | 438 | HFSSM | +7/9 |
|  |  | Wolde et al. | 2015 | Cross-sectional | 450 | HFIAS | +7/9 |
| Stunting | [12, 39-43, 45-57] | Osei et al. | 2010 | Cross-sectional | 368 | HFIAS | +7/9 |
|  |  | Betebo et al. | 2017 | Cross-sectional | 508 | HFIAS | +7/9 |
|  |  | Isanaka et al. | 2007 | Cross-sectional | 2526 | HFSSM | +8/9 |
|  |  | Naser et al. | 2014 | Cross-sectional | 233 | Radimer/Cornell Hunger and Food Insecurity instrument | +6/9 |
|  |  | Shahraki et al. | 2016 | Cross-sectional | 610 | HFSSM | +7/9 |
|  |  | Singh et al. | 2014 | Cross-sectional | 235 | HFIAS | +6/9 |
|  |  | Wolde et al. | 2015 | Cross-sectional | 450 | HFIAS | +7/9 |
|  |  | Belayne et al. | 2021 | Cohort | 935 | HFIAS | +8/9 |
|  |  | Cook et al. | 2006 | Cohort | 17130 | HFSSM | +9/9 |
|  |  | Hagos et al. | 2017 | Cross-sectional | 4094 | HFIAS | +8/9 |
|  |  | Kabalo et al. | 2022 | Cohort | 907 | HFIAS | +8/9 |
|  |  | Koyratti et al. | 2022 | Cohort | 1166 | Local survey | +8/9 |
|  |  | Mahmudiono et al. | 2018 | Cross-sectional | 685 | HFIAS | +7/9 |
|  |  | McDonald et al. | 2014 | Cross-sectional | 900 | HFIAS | +7/9 |
|  |  | Mutisya et al. | 2015 | Cross-sectional | 6858 | Radimer/Cornell Hunger and Food Insecurity instrument | +8/9 |
|  |  | Na et al. | 2020 | Cohort | 12693 | a 9- item Food Access Survey Tool (FAST), | +9/9 |
|  |  | Namirembe et al. | 2022 | Cohort | 4528 | HFIAS | +8/9 |
|  |  | Nkurunziza et al. | 2017 | Cross-sectional | 6199 | HFIAS | +8/9 |
|  |  | Shinsugi et al. | 2015 | Cross-sectional | 404 | HFIAS | +7/9 |
| Wasting | [39, 41, 42, 45, 52] | Betebo et al. | 2017 | Cross-sectional | 508 | HFIAS | +7/9 |
|  |  | Naser et al. | 2014 | Cross-sectional | 233 | Radimer/Cornell Hunger and Food Insecurity instrument | +6/9 |
|  |  | Shahraki et al. | 2016 | Cross-sectional | 610 | HFSSM | +7/9 |
|  |  | Wolde et al. | 2015 | Cross-sectional | 450 | HFIAS | +7/9 |
|  |  | McDonald et al. | 2014 | Cross-sectional | 900 | HFIAS | +7/9 |
| Dental caries | [58-67] | Angelopoulou et al. | 2019 | Cross-sectional | 82 | HFSSM | +6/9 |
|  |  | Bae et al. | 2018 | Cross-sectional | 2950 | NR | +7/9 |
|  |  | Bahanan et al. | 2021 | Cross-sectional | 10723 | HFSSM | +8/9 |
|  |  | Braunstein et al. | 2008 | Cross-sectional | 801 (2-5 years)  1097 (6-11 years) | HFSSM | +8/9 |
|  |  | Chi et al. | 2014 | Cross-sectional | 2206 | HFSSM | +8/9 |
|  |  | Ferreira et al. | 2019 | Cross-sectional | 538 | Brazilian Food Insecurity Scale | +7/9 |
|  |  | Frazao et al. | 2014 | Cross-sectional | 203 | Brazilian Food Insecurity Scale | +6/9 |
|  |  | Jackson et al. | 2021 | Cross-sectional | 99961 | Food insecurity was assessed using a four questions. | +8/9 |
|  |  | Santin et al. | 2016 | Cross-sectional | 538 | Brazilian Food Insecurity Scale | +7/9 |
|  |  | Tsai et al. | 2021 | Cross-sectional | 229 (On-reserve  Population)  115 (Off-reserve  population) | Food insecurity was assessed using a single question. | +6/9 |
| Suicidal behaviors (adolescents) | [68-95] | Alaimo et al. | 2002 | Cross-sectional | 754 | Food insecurity was assessed using a single question. | +8/9 |
|  |  | Almansour et al. | 2017 | Cross-sectional | 3680 | NR | +7/9 |
|  |  | Altangerel et al. | 2014 | Cross-sectional | 5191 | NR | +7/9 |
|  |  | Arat et al. | 2017 | Cross-sectional | 3215 (Uganda)  3691 (Kenya)  2257 (Zambia)  2197 (Botswana)  2176 (Tanzania)  1432 (Seychelles) | Food insecurity was assessed using a three questions. | +8/9 |
|  |  | Oppong Asante et al. | 2017 | Cross-sectional | 1984 | Food insecurity was assessed using a single question. | +8/9 |
|  |  | Dema et al. | 2019 | Cross-sectional | 5809 | self-administered questionnaire | +7/9 |
|  |  | Kwangu et al. | 2017 | Cross-sectional | 5192 (Pakistan) | NR | +7/9 |
|  |  | Mazaba et al. | 2017 | Cross-sectional | 1908 | NR | +7/9 |
|  |  | Mazaba et al. | 2017 | Cross-sectional | 1673 | NR | +7/9 |
|  |  | Mazaba et al. | 2017 | Cross-sectional | 2672 | NR | +7/9 |
|  |  | Mazaba et al. | 2017 | Cross-sectional | 3331 | NR | +7/9 |
|  |  | Mulenga et al. | 2017 | Cross-sectional | 2027 | NR | +7/9 |
|  |  | Mulenga et al. | 2017 | Cross-sectional | 2607 | NR | +7/9 |
|  |  | Mulenga et al. | 2017 | Cross-sectional | 2811 | NR | +7/9 |
|  |  | Kwangu et al. | 2017 | Cross-sectional | 5192 | NR | +7/9 |
|  |  | Kwangu et al. | 2017 | Cross-sectional | 1648 | NR | +7/9 |
|  |  | Nyundo et al. | 2020 | Cross-sectional | 7662 (Ethiopia)  7662 (Nigeria) | 5-point Likert scale | +8/9 |
|  |  | Pandey et al. | 2019 | Cross-sectional | 6531 | Food insecurity was assessed using a single questions adapted from the HFFSM. | +8/9 |
|  |  | Pengpid et al. | 2020 | Cross-sectional | 2744 | Food insecurity was assessed using a single question. | +8/9 |
|  |  | Romo et al. | 2016 | Cross-sectional | 5524 | Food insecurity was assessed using a single question. | +8/9 |
|  |  | Shayo et al. | 2019 | Cross-sectional | 3793 | Food insecurity was assessed using a single question. | +8/9 |
|  |  | Siziya et al. | 2017 | Cross-sectional | 2418 | NR | +7/9 |
|  |  | Siziya et al. | 2017 | Cross-sectional | 1623 | NR | +7/9 |
|  |  | Teevale et al. | 2016 | Cross-sectional | 1445 | NR | +7/9 |
|  |  | Ziaei et al. | 2017 | Cross-sectional | 1517 | self-administered questionnaire | +7/9 |
|  |  | Mulenga et al. | 2017 | Cross-sectional | 2924 | NR | +7/9 |
|  |  | Nii-Boye Quarshie et al. | 2022 | Cross-sectional | 1437 | NR | +7/9 |
|  |  | Njunju et al. | 2017 | Cross-sectional | 2803 | NR | +7/9 |
| Early childhood development disorders (children) | [22, 96-108] | Drennen et al. | 2019 | Cross-sectional | 28184 | HFSSM | +8/9 |
|  |  | Black et al | 2012 | Cross-sectional | 26950 | HFSSM | +8/9 |
|  |  | Finch et al. | 2018 | Cohort | 1302 | HFIAS | +9/9 |
|  |  | Gill et al. | 2018 | Cross-sectional | 4125 | HFSSM | +8/9 |
|  |  | Herandez et al. | 2009 | Cohort | 7900 | HFSSM | +9/9 |
|  |  | hobbs et al. | 2018 | Cohort | 2046 | HFSSM | +9/9 |
|  |  | Huang et al. | 2018 | Cohort | 8900 | HFSSM | +9/9 |
|  |  | Johnson et al. | 2018 | Cohort | 3700 | HFSSM | +8/9 |
|  |  | Mikens et al. | 2019 | Cohort | 792 | NR | +7/9 |
|  |  | Milner et al. | 2018 | Cohort | 304 | HFIAS | +7/9 |
|  |  | Nagata et al. | 2018 | Cohort | 168 | HFSSM | +8/9 |
|  |  | Obradovic et al. | 2016 | Cohort | 8944 | HFIAS | +9/9 |
|  |  | Rose-Jacobs et al. | 2008 | Cross-sectional | 2010 | HFSSM | +8/9 |
| Abbreviations: ELCSA: Latin American and Caribbean Food Security Scale, HFIAS: Household Food Insecurity Access Scale, HFSSM: The Household Food Security Survey Module, USFSSM: U.S. Household Food Security Survey Module, NR: Not Report | | | | | | | |

Supplementary Table 5. GRADE evidence table for the association between food insecurity and adverse health outcomes in children and adolescents

**Question**: Is there an association between food insecurity and adverse health outcomes among children and adolescents?

**Setting**: Children and adolescents

| **Outcomes** | **Certainty assessment** | | | | | | | **Effect** | **Certainty** | **Importance** |
| --- | --- | --- | --- | --- | --- | --- | --- | --- | --- | --- |
| **Adverse health outcomes** | **№ of studies** | **Study design** | **Risk of bias** | **Inconsistency** | **Indirectness** | **Imprecision** | **Publication bias** | **Mean difference (95%CI)** |  |  |
| Anemia | 19 | Cross-sectional | not serious | serious | not serious | not serious | not serious | 1.50 (1.23, 1.82) | ⨁⨁⨁◯ Moderate | IMPORTANT |
| Obesity | 21 | Cross-sectional | not serious | not serious | serious | not serious | not serious | 1.16 (1.06, 1.27) | ⨁⨁⨁◯ Moderate | IMPORTANT |
| Overweight | 11 | Cross-sectional | not serious | serious | serious | serious | not serious | 1.08 (0.99, 1.18) | ⨁◯◯◯ Very LOW | IMPORTANT |
| Stunning | 19 | Cross-sectional | not serious | serious | not serious | not serious | not serious | 1.12 (1.05, 1.19) | ⨁⨁⨁◯ Moderate | IMPORTANT |
| Underweight | 13 | Cross-sectional | not serious | serious | not serious | serious | not serious | 1.16 (0.98, 1.38) | ⨁⨁◯◯ LOW | IMPORTANT |
| Wasting | 5 | Cross-sectional | not serious | not serious | not serious | serious | not serious | 1.04 (0.96, 1.13) | ⨁⨁⨁◯ Moderate | IMPORTANT |
| Dental caries | 16 | Cross-sectional | not serious | serious | serious | not serious | not serious | 1.70 (1.43, 2.03) | ⨁⨁◯◯ LOW | IMPORTANT |
| Suicide ideation | 30 | Cross-sectional | not serious | serious | not serious | not serious | not serious | 1.08 (1.01, 1.16) | ⨁⨁⨁◯ Moderate | IMPORTANT |
| Suicide attempt | 18 | Cross-sectional | not serious | serious | not serious | serious | not serious | 1.05 (0.91, 1.22) | ⨁⨁◯◯ LOW | IMPORTANT |
| Suicide plan | 11 | Cross-sectional | not serious | serious | not serious | not serious | not serious | 1.32 (1.14, 1.54) | ⨁⨁⨁◯ Moderate | IMPORTANT |
| Unspecified suicide | 2 | Cross-sectional | not serious | serious | not serious | serious | not serious | 1.54 (0.60, 3.98) | ⨁⨁◯◯ LOW | IMPORTANT |
| Developmental risk | 3 | Cross-sectional | not serious | not serious | serious | not serious | not serious | 1.32 (1.10, 1.59) | ⨁⨁⨁◯ Moderate | IMPORTANT |
| Motor | 2 | Cross-sectional | not serious | not serious | not serious | serious | not serious | 0.91 (0.80, 1.04) | ⨁⨁⨁◯ Moderate | IMPORTANT |
| Cognitive/vocabulary | 2 | Cross-sectional | not serious | not serious | serious | serious | not serious | 0.92 (0.83, 1.02) | ⨁⨁◯◯ LOW | IMPORTANT |
| Cognitive/math | 3 | Cross-sectional | not serious | not serious | serious | serious | not serious | 0.84 (0.73, 0.96) | ⨁⨁◯◯ LOW | IMPORTANT |
| Cognitive/school readiness and reading | 3 | Cross-sectional | not serious | not serious | serious | serious | not serious | 0.91 (0.82, 1.00) | ⨁⨁◯◯ LOW | IMPORTANT |
| Externalizing behavior | 6 | Cross-sectional | not serious | serious | serious | not serious | not serious | 1.25 (1.08, 1.44) | ⨁⨁◯◯ LOW | IMPORTANT |
| Externalizing behavior/hyperactivity | 3 | Cross-sectional | not serious | serious | serious | serious | not serious | 1.18 (0.79, 1.76) | ⨁◯◯◯ Very LOW | IMPORTANT |
| Internalizing behavior | 2 | Cross-sectional | not serious | serious | serious | serious | not serious | 1.48 (0.70, 3.15) | ⨁◯◯◯ Very LOW | IMPORTANT |
| Internalizing behavior/anxiety | 3 | Cross-sectional | not serious | serious | serious | serious | not serious | 1.14 (0.82, 1.57) | ⨁◯◯◯ Very LOW | IMPORTANT |

| **Supplementary Table 6**. Meta-regression analysis results for confounding variables | | | | |
| --- | --- | --- | --- | --- |
|  | Race/Ethnicity | Sex | Age | Food insecurity assessment tool |
| **Adverse health outcomes** | β (95% CI), P-value | β (95% CI), P-value | β (95% CI), P-value | β (95% CI), P-value |
| Anemia | -1.49 (-2.43 to -0.55), 0.004 | -0.28 (-0.64 to -0.07), 0.114 | -0.25 (-1.19 to 0.69), 0.580 | 1.08 (0.11 to 2.05), 0.031 |
| Obesity | 0.06 (-1.02 to 1.14), 0.909 | 0.0 (-0.37 to 0.37), 0.993 | 0.91 (-0.62 to 2.46), 0.228 | 0.40 (-1.42 to 2.23), 0.648 |
| Stunning | 0.09 (-1.77 to -1.97), 0.913 | 0.10 (-0.22 to 0.22), 1.00 | 0.70 (-0.42 to 1.83), 0.205 | -0.29 (-2.06 to 1.47), 0.729 |
| Dental caries | 0.08 (-0.63 to 0.81), 0.795 | 0.0 (-0.53 to 0.53), 1.00 | -0.67 (-1.37 to 0.02), 0.058 | 0.46 (-0.57 to 1.50), 0.357 |
| Underweight | 0.10 (-1.33 to 1.53), 0.879 | 0.0 (-0.31 to 0.31), 1.00 | 0.09 (-0.82 to 1.01), 0.826 | -0.30 (-0.83 to 1.44), 0.567 |
| Overweight | 0.03 (-1.15 to 1.21), 0.952 | 0.0 (-0.44 to 0.44), 1.00 | 1.59 (-1.09 to 4.28), 0.213 | -0.79 (-0.46 to 3.02), 0.650 |
| Suicide ideation | 0.01 (-0.92 to 0.95), 0.980 | 0.0 (0.01 to 0.01), 1.0 | 0.0 (0.01 to 0.01), 1.0 | 0.0 (0.01 to 0.01), 1.0 |
| Suicide plan | 0.17 (-0.50 to 0.85), 0.565 | 0.0 (-0.06 to 0.06), 1.00 | 0.0 (-0.06 to 0.06), 1.00 | 0.0 (-0.06 to 0.06), 1.00 |
| Suicide attempt | -0.02 (-0.52 to 0.47), 0.906 | 0.0 (-0.05 to 0.05), 1.00 | 0.0 (-0.05 to 0.05), 1.00 | 0.0 (-0.05 to 0.05), 1.00 |

| **Supplementary Table 7.**  Methodological quality of included systematic reviews using AMSTAR 2. | | | | | | | | | | | | | | | | | |  |
| --- | --- | --- | --- | --- | --- | --- | --- | --- | --- | --- | --- | --- | --- | --- | --- | --- | --- | --- |
| **Author, year (ref.)** | **Q1** | **Q2** | **Q3** | **Q4** | **Q5** | **Q6** | **Q7** | **Q8** | **Q9** | **Q10** | **Q11** | **Q12** | **Q13** | **Q14** | **Q15** | **Q16** | **Quality of evidence** | |
| Belachew et al, 2020[109] | **Y** | **N** | **Y** | **Y** | **Y** | **Y** | **PY** | **Y** | **Y** | **N** | **Y** | **Y** | **Y** | **Y** | **Y** | **Y** | **Low** | |
| Derakhshandeh-Rishehri et al, 2021 [110] | **Y** | **N** | **Y** | **Y** | **Y** | **Y** | **PY** | **Y** | **Y** | **N** | **Y** | **Y** | **Y** | **Y** | **Y** | **Y** | **Low** | |
| Drumond et al, 2023[111] | **Y** | **Y** | **Y** | **Y** | **Y** | **Y** | **Y** | **Y** | **Y** | **N** | **Y** | **Y** | **Y** | **Y** | **Y** | **Y** | **High** | |
| Eskandari et al, 2022[112] | **Y** | **Y** | **Y** | **Y** | **Y** | **Y** | **Y** | **Y** | **Y** | **Y** | **Y** | **Y** | **N** | **Y** | **N** | **Y** | **Critically Low** | |
| Kaggwa et al, 2023[113] | **N** | **Y** | **Y** | **Y** | **Y** | **Y** | **Y** | **Y** | **Y** | **N** | **Y** | **Y** | **Y** | **Y** | **Y** | **Y** | **Moderate** | |
| Moradi et al, 2018[114] | **Y** | **N** | **Y** | **Y** | **Y** | **Y** | **Y** | **Y** | **Y** | **N** | **Y** | **Y** | **Y** | **Y** | **Y** | **Y** | **Low** | |
| Moradi et al, 2019[115] | **Y** | **N** | **Y** | **Y** | **Y** | **Y** | **Y** | **Y** | **Y** | **N** | **Y** | **Y** | **Y** | **Y** | **Y** | **N** | **Low** | |
| de Oliveira et al, 2020[116] | **Y** | **Y** | **Y** | **Y** | **Y** | **Y** | **PY** | **Y** | **Y** | **Y** | **Y** | **Y** | **N** | **Y** | **N** | **Y** | **Critically Low** | |
| Patriota et al, 2024[117] | **Y** | **Y** | **Y** | **Y** | **Y** | **Y** | **Y** | **Y** | **Y** | **Y** | **Y** | **Y** | **N** | **Y** | **N** | **Y** | **Critically Low** | |
| Pourmotabbed et al, 2020[118] | **Y** | **N** | **Y** | **Y** | **Y** | **Y** | **Y** | **Y** | **Y** | **N** | **Y** | **Y** | **Y** | **Y** | **Y** | **Y** | **Low** | |
| Sabbagh et al, 2023[119] | **Y** | **Y** | **Y** | **Y** | **Y** | **Y** | **Y** | **Y** | **Y** | **Y** | **Y** | **Y** | **Y** | **Y** | **Y** | **Y** | **High** | |
| Zhou et al, 2023[120] | **Y** | **N** | **Y** | **Y** | **N** | **Y** | **Y** | **Y** | **Y** | **N** | **Y** | **Y** | **Y** | **Y** | **Y** | **Y** | **Low** | |

ref, reference; PY, partially yes. Q1: Did the research questions and inclusion criteria for the review include the components of PICO?, Q2: 2. Did the report of the review contain an explicit statement that the review methods were established prior to the conduct of the review and did the report justify any significant deviations from the protocol?; Q3, Did the review authors explain their selection of the study designs for inclusion in the review?; Q4, Did the review authors use a comprehensive literature search strategy?; Q5, Did the review authors perform study selection in duplicate?; Q6, Did the review authors perform data extraction in duplicate?; Q7, Did the review authors provide a list of excluded studies and justify the exclusions?; Q8, Did the review authors describe the included studies in adequate detail?; Q9, Did the review authors use a satisfactory technique for assessing the risk of bias?; Q10, Did the review authors report on the sources of funding?; Q11, Did the review authors use appropriate methods for statistical combination of results?; Q12, Did the review authors assess the potential impact of RoB in individual studies on the results?; Q13, Did the review authors account for RoB in individual studies when interpreting/ discussing the results of the review?; Q14, Did the review authors provide a satisfactory explanation for, and discussion of, any heterogeneity?; Q15, Did the review authors carry out an adequate investigation

**
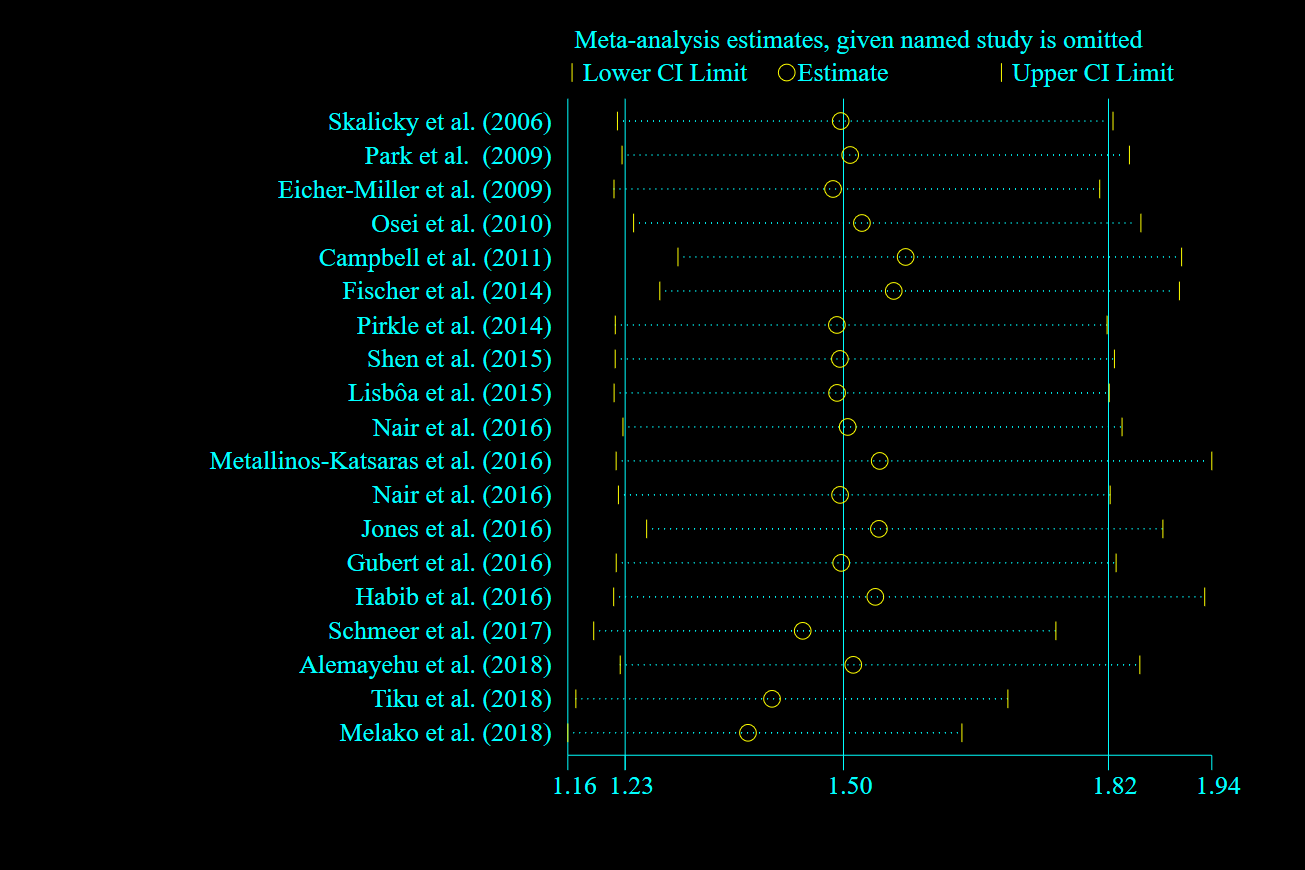
**

**Supplementary Figure 1-A.** Forest plots showing sensitivity analysis results of the relationship between food insecurity and risk of anemia.


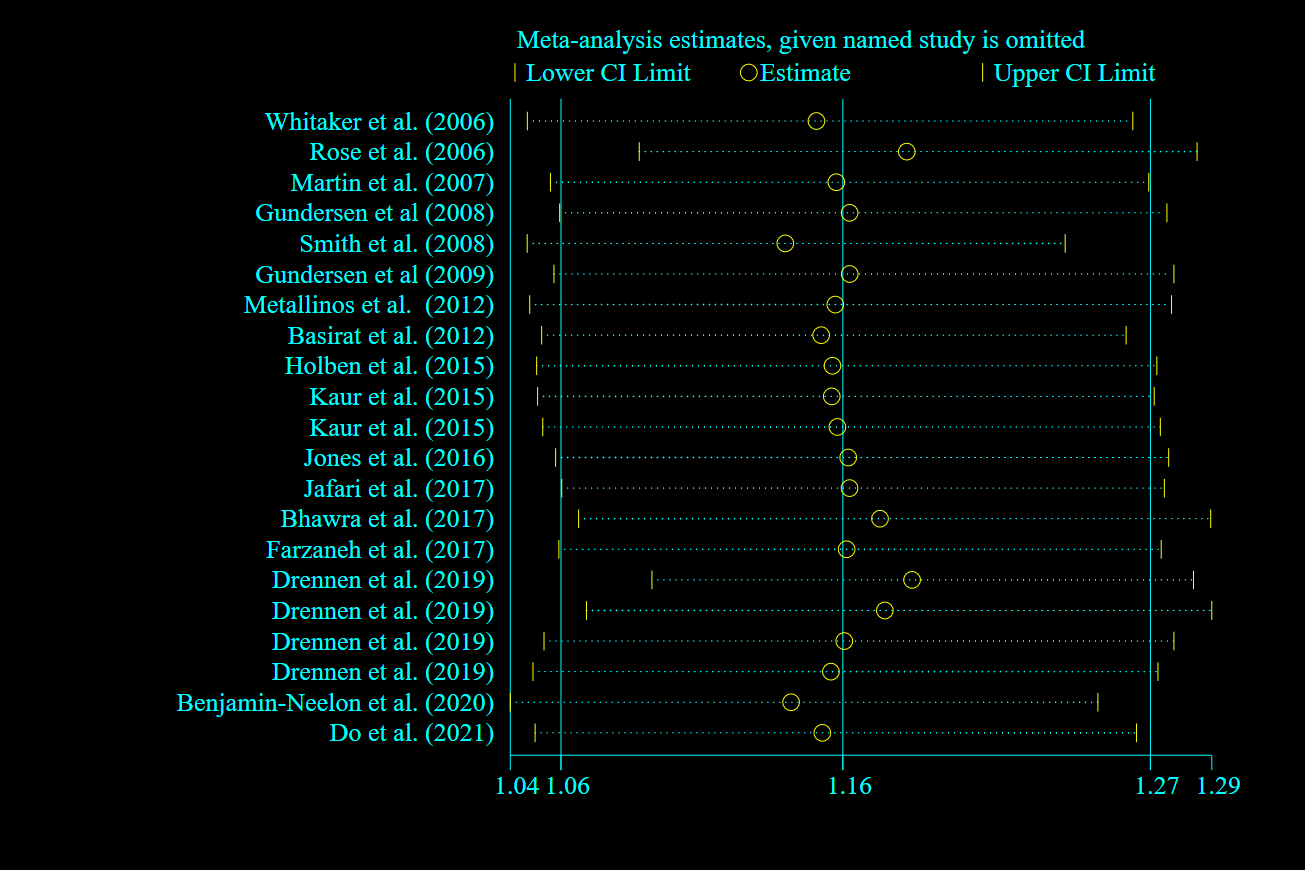


**Supplementary Figure 1-B.** Forest plots showing sensitivity analysis results of the relationship between food insecurity and risk of obesity.


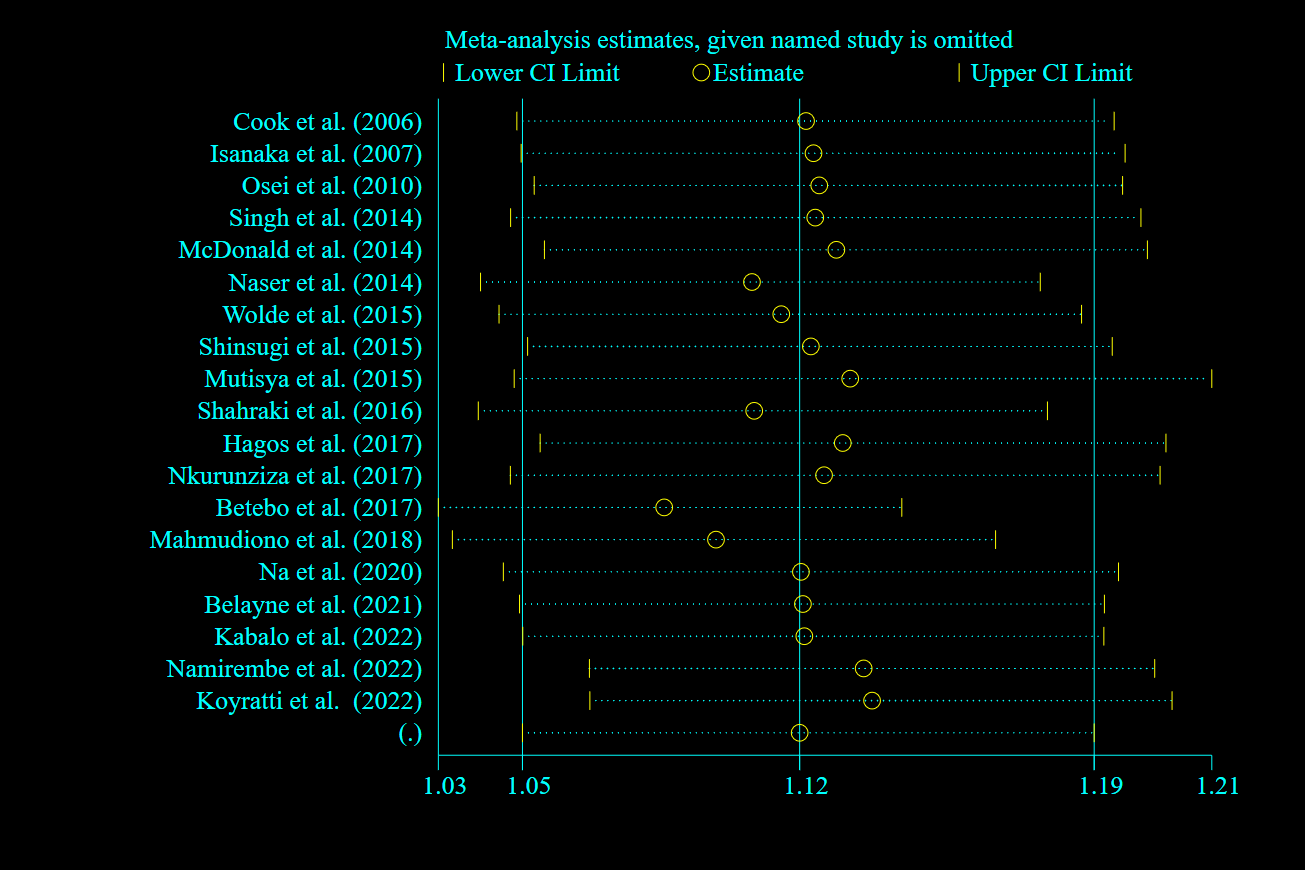


**Supplementary Figure 1-C.** Forest plots showing sensitivity analysis results of the relationship between food insecurity and risk of stunning.


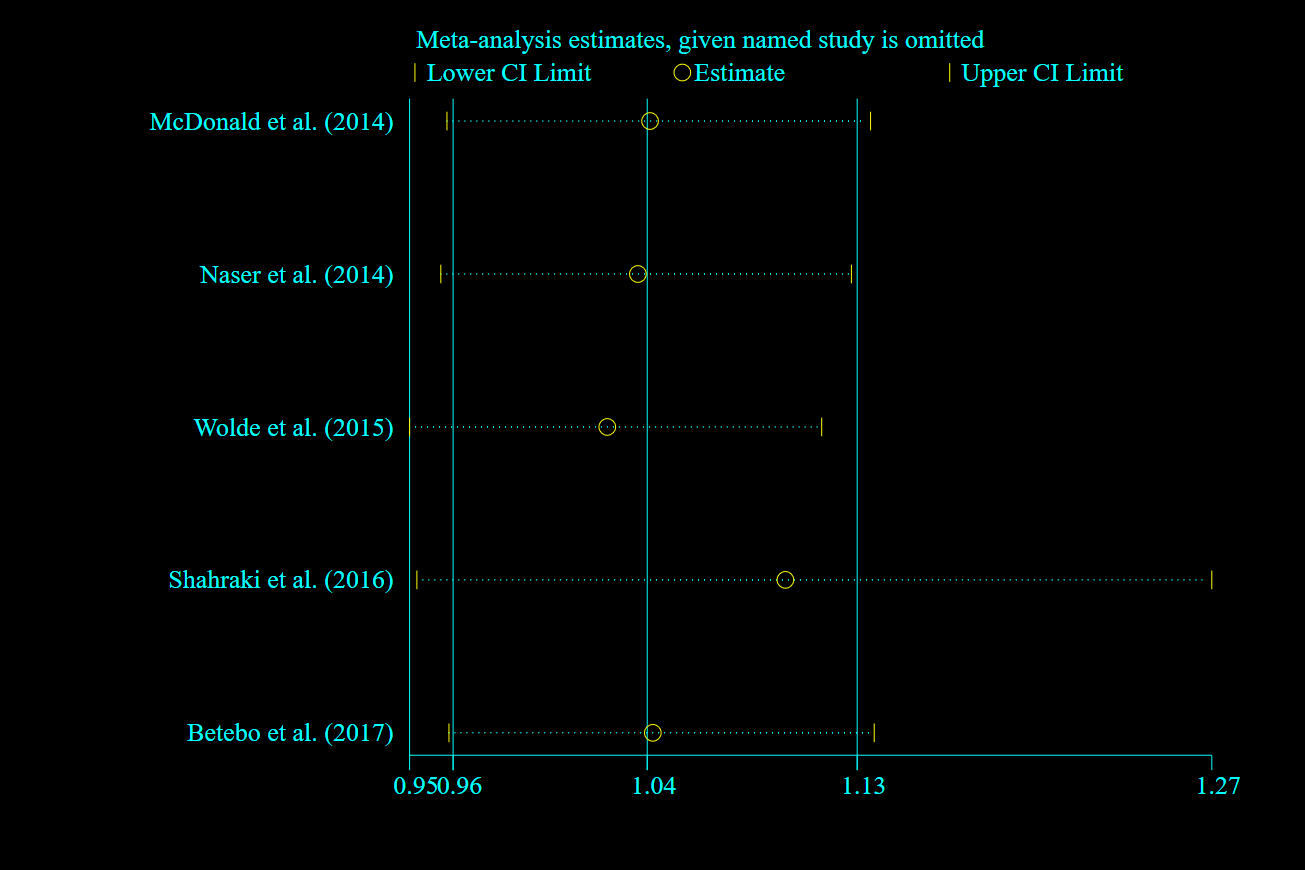


**Supplementary Figure 1-D.** Forest plots showing sensitivity analysis results of the relationship between food insecurity and risk of wasting.


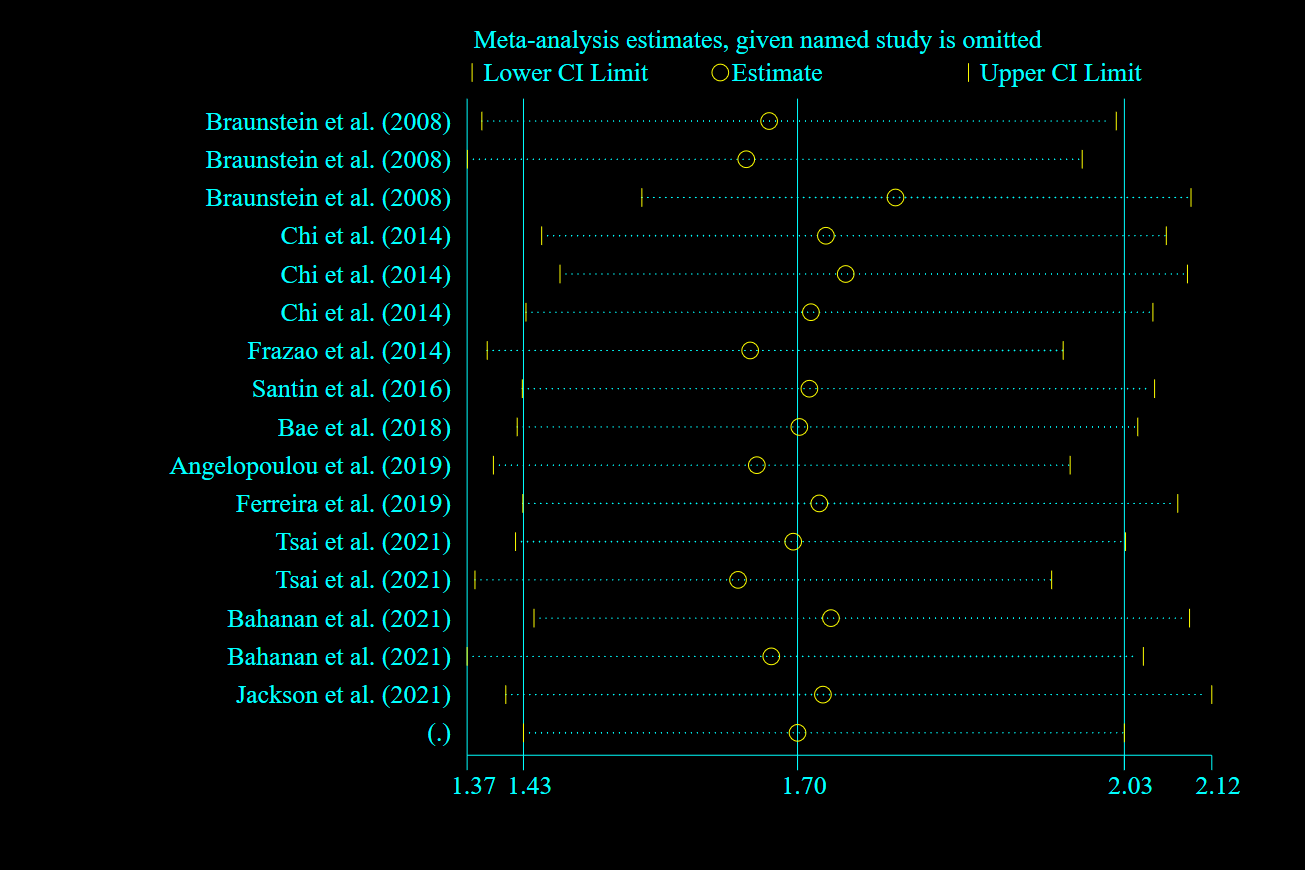


**Supplementary Figure 1-E.** Forest plots showing sensitivity analysis results of the relationship between food insecurity and risk of dental caries.


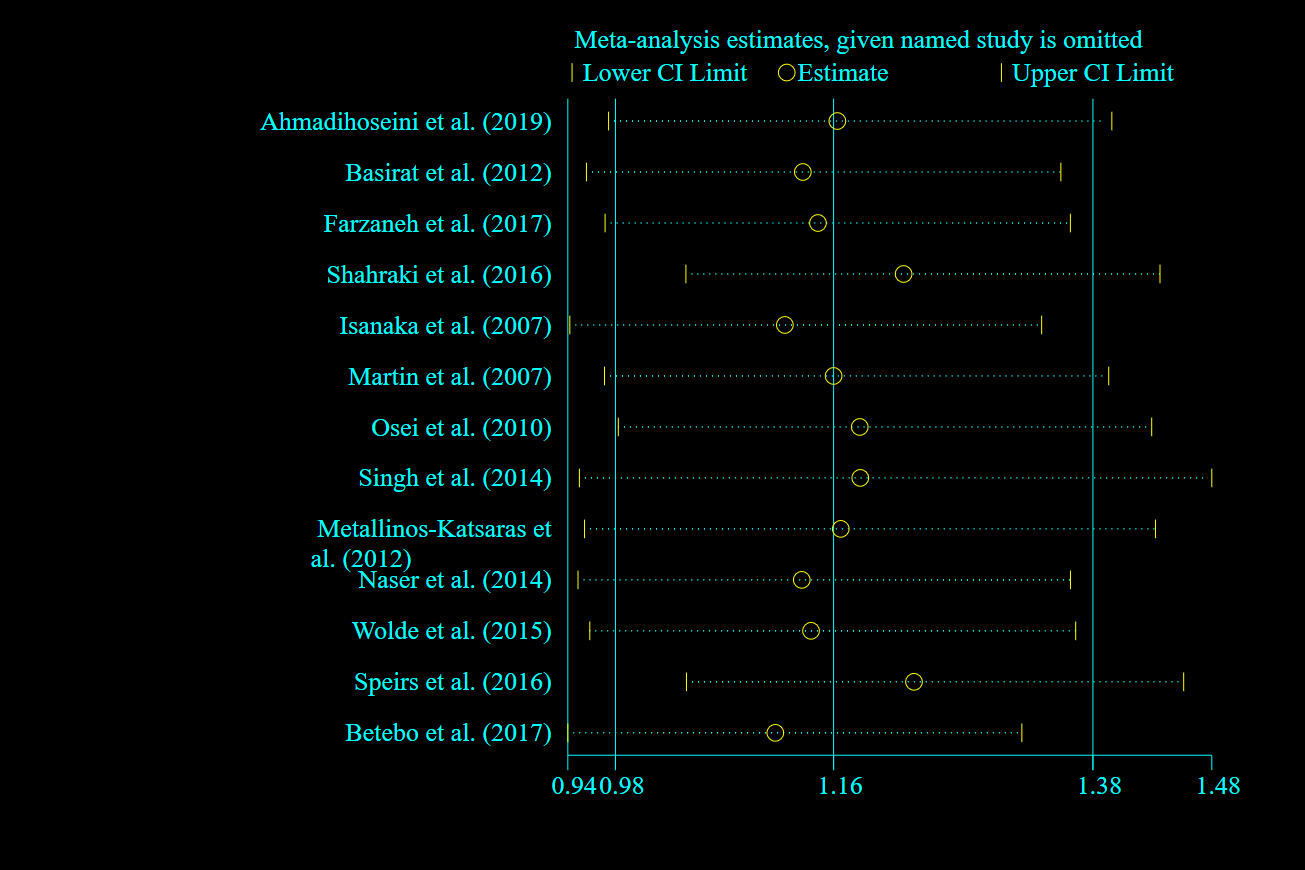


**Supplementary Figure 1-F.** Forest plots showing sensitivity analysis results of the relationship between food insecurity and risk of underweight.

**
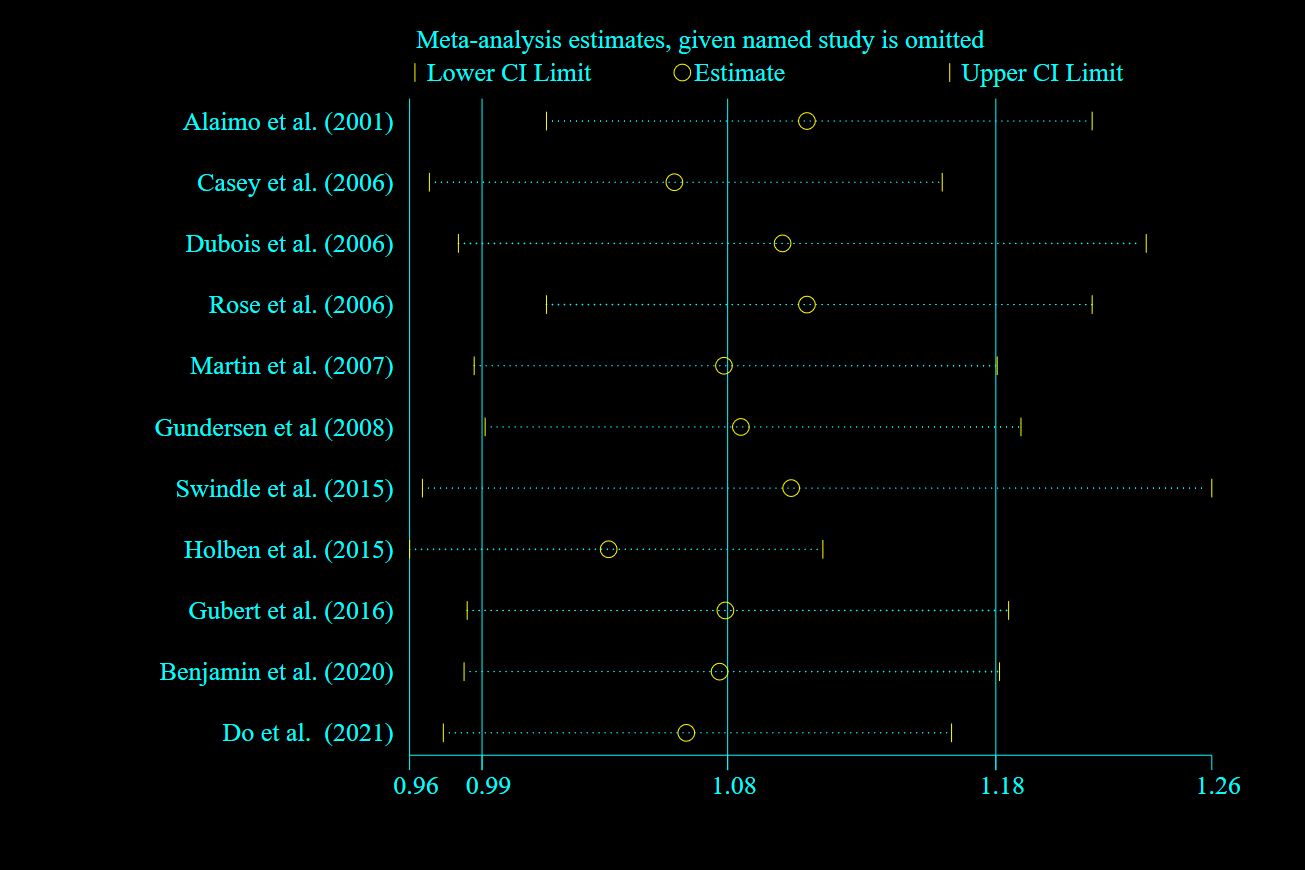
**

**Supplementary Figure 1-G.** Forest plots showing sensitivity analysis results of the relationship between food insecurity and risk of overweight.

**
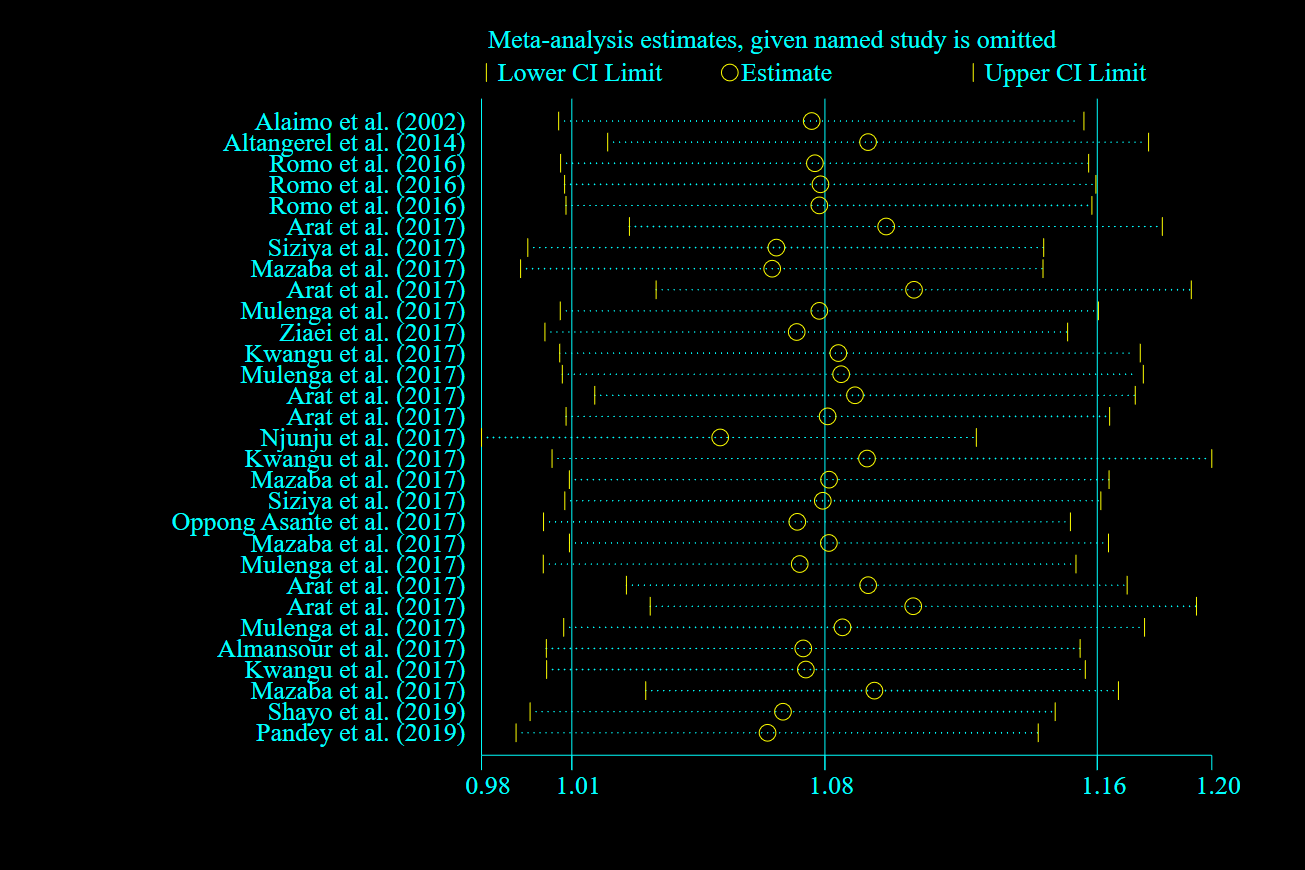
**

**Supplementary Figure 1-H.** Forest plots showing sensitivity analysis results of the relationship between food insecurity and risk of suicide ideation.


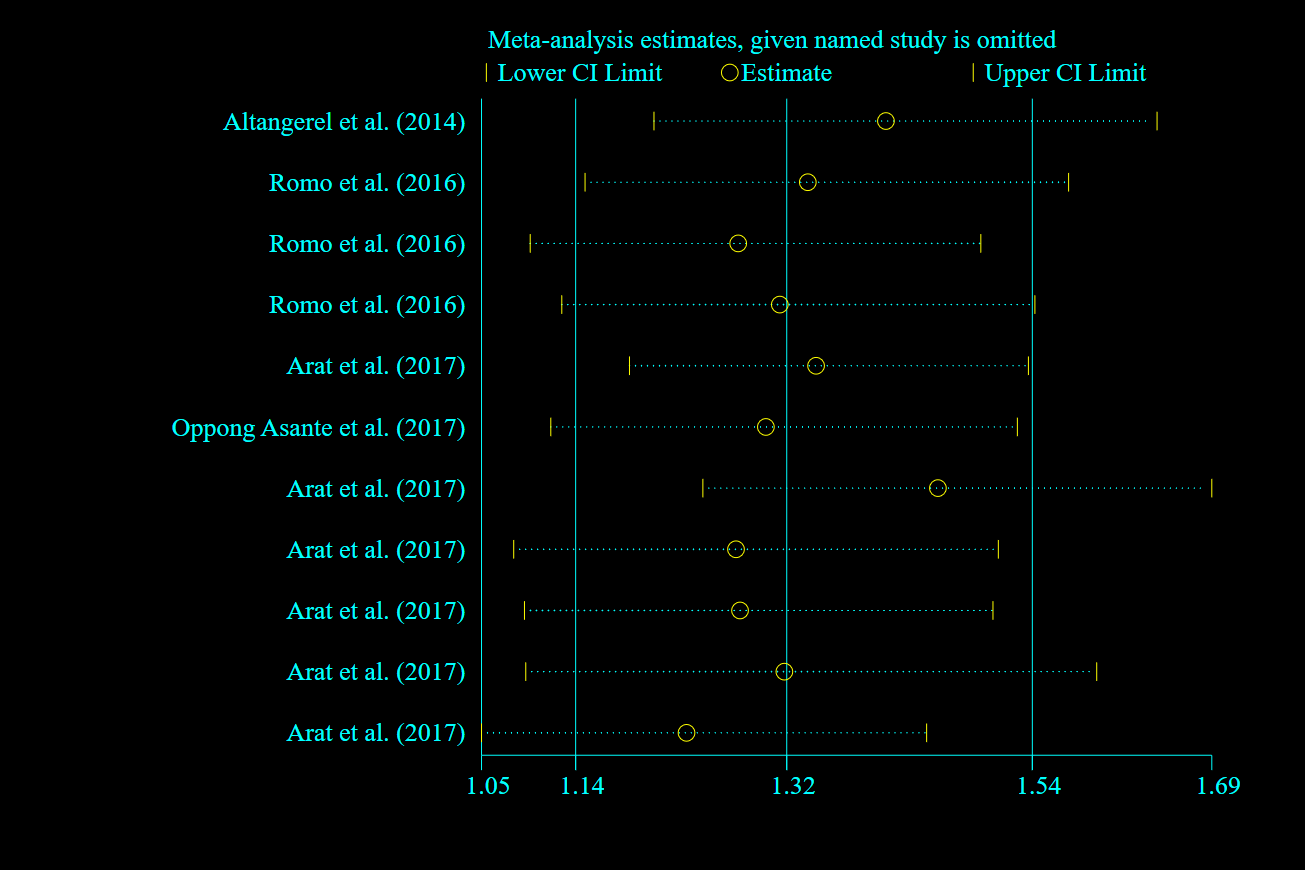


**Supplementary Figure 1-I.** Forest plots showing sensitivity analysis results of the relationship between food insecurity and risk of suicide plan.


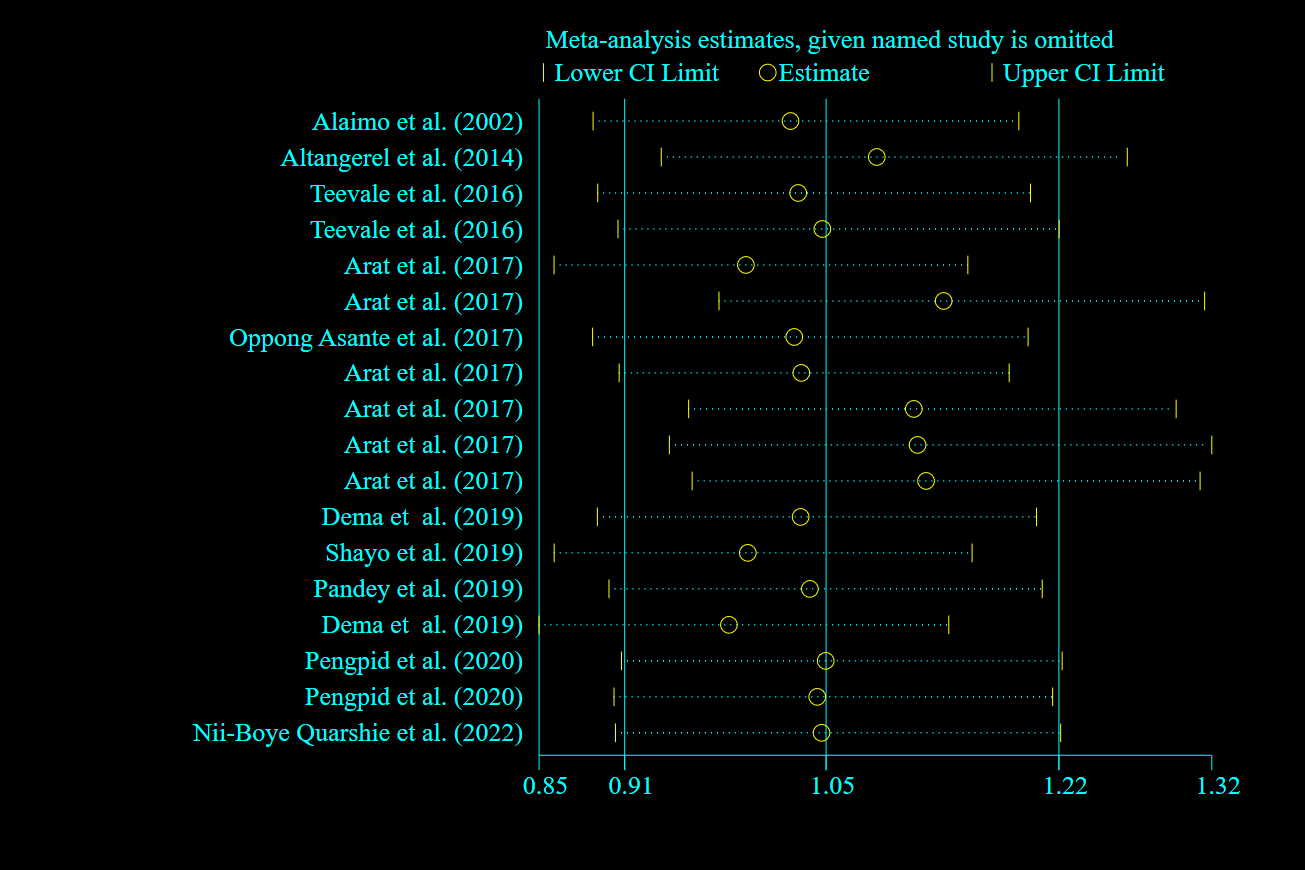


**Supplementary Figure 1-J.** Forest plots showing sensitivity analysis results of the relationship between food insecurity and risk of suicide attempt.


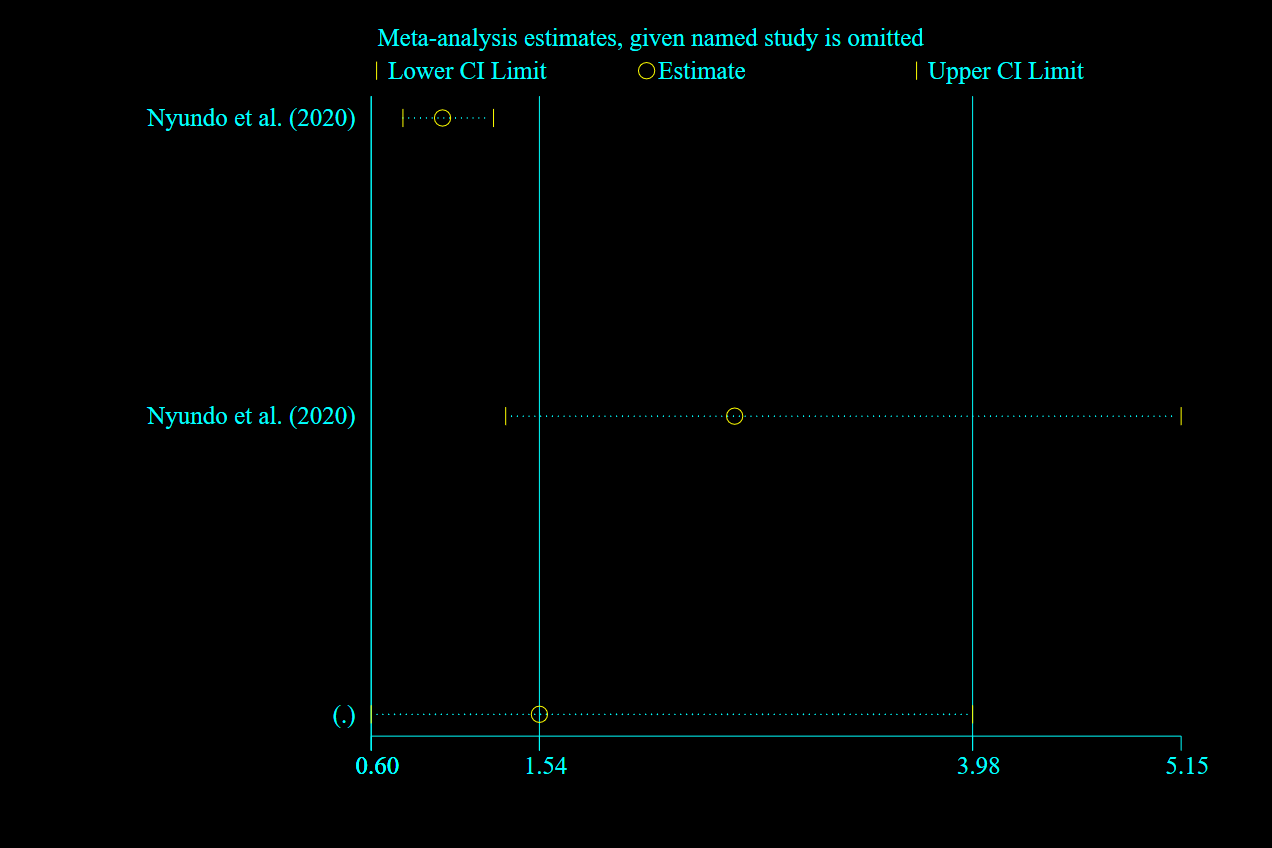


**Supplementary Figure 1-K.** Forest plots showing sensitivity analysis results of the relationship between food insecurity and risk of unspecified suicide.


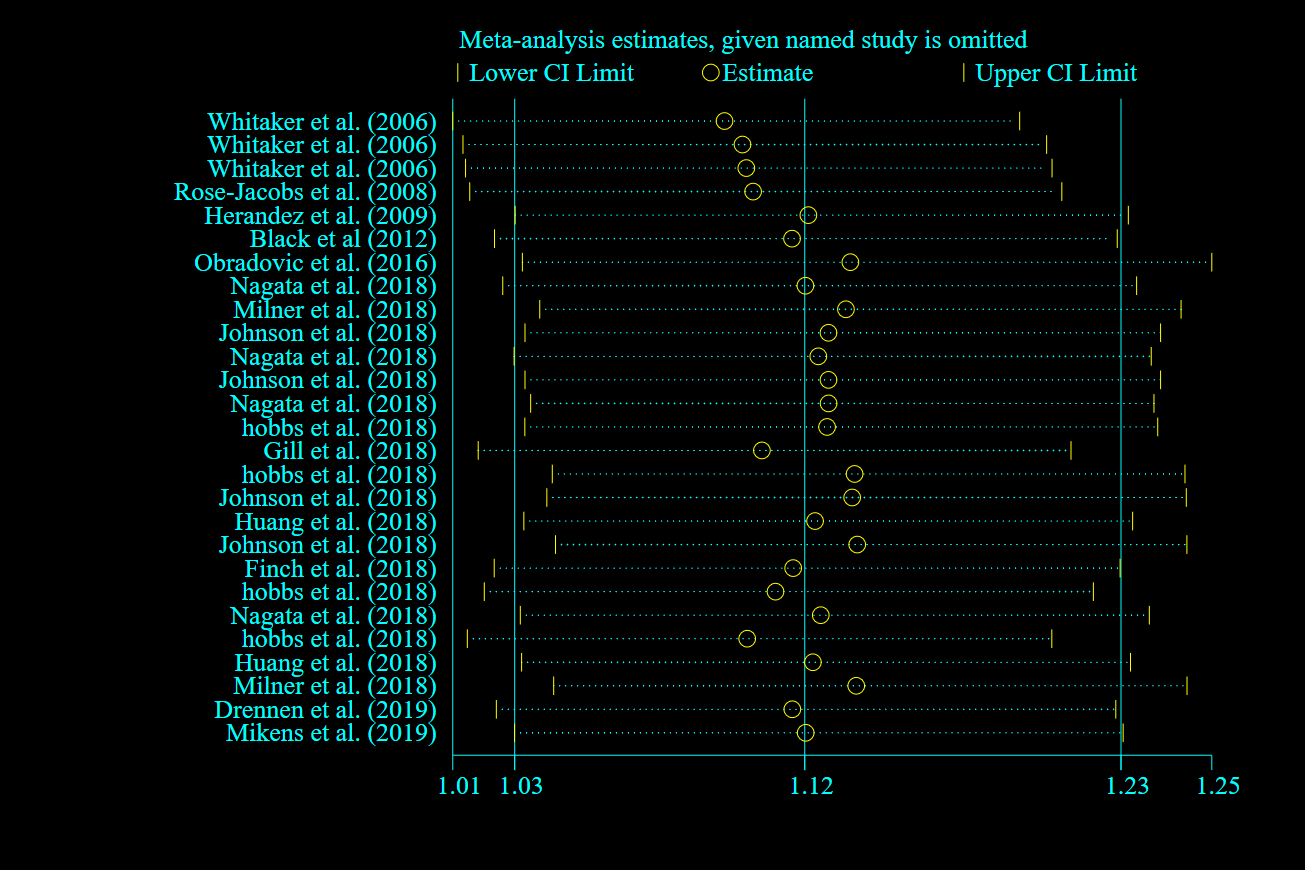


**Supplementary Figure 1-M.** Forest plots showing sensitivity analysis results of the relationship between food insecurity and risk of early childhood development disorders.

**Additional references:**

1. Alemayehu, M., et al., *Prevalence and correlates of anemia among children aged 6-23 months in Wolaita Zone, Southern Ethiopia.* PloS one, 2019. **14**(3): p. e0206268.

2. Campbell, A., et al., *Relationship of household food insecurity to anaemia in children aged 6–59 months among families in rural Indonesia.* Annals of tropical paediatrics, 2011. **31**(4): p. 321-330.

3. Eicher-Miller, H.A., et al., *Food insecurity is associated with iron deficiency anemia in US adolescents.* The American journal of clinical nutrition, 2009. **90**(5): p. 1358-1371.

4. Fischer, N.C., et al., *Household food insecurity is associated with anemia in adult Mexican women of reproductive age.* The Journal of nutrition, 2014. **144**(12): p. 2066-2072.

5. Gubert, M.B., et al., *Household food insecurity, nutritional status and morbidity in Brazilian children.* Public Health Nutrition, 2016. **19**(12): p. 2240-2245.

6. Habib, M.A., et al., *Prevalence and predictors of iron deficiency anemia in children under five years of age in Pakistan, a secondary analysis of national nutrition survey data 2011–2012.* PloS one, 2016. **11**(5): p. e0155051.

7. Jones, A.D., et al., *Household food insecurity in Mexico is associated with the co‐occurrence of overweight and anemia among women of reproductive age, but not female adolescents.* Maternal & Child Nutrition, 2017. **13**(4): p. e12396.

8. Lisbôa, M.B.M.d.C., et al., *Prevalence of iron-deficiency anemia in children aged less than 60 months: A population-based study from the state of Minas Gerais, Brazil.* Revista de Nutrição, 2015. **28**(2): p. 121-131.

9. Malako, B.G., M.S. Teshome, and T. Belachew, *Anemia and associated factors among children aged 6–23 months in Damot Sore District, Wolaita Zone, South Ethiopia.* BMC hematology, 2018. **18**: p. 1-9.

10. Metallinos-Katsaras, E., et al., *Household food security status is associated with anemia risk at age 18 months among low-income infants in Massachusetts.* Journal of the Academy of Nutrition and Dietetics, 2016. **116**(11): p. 1760-1766.

11. Nair, K.M., et al., *Characterisation of anaemia and associated factors among infants and pre-schoolers from rural India.* Public health nutrition, 2016. **19**(5): p. 861-871.

12. Osei, A., et al., *Household food insecurity and nutritional status of children aged 6 to 23 months in Kailali District of Nepal.* Food and nutrition bulletin, 2010. **31**(4): p. 483-494.

13. Park, K., et al., *Household food insecurity is a risk factor for iron-deficiency anaemia in a multi-ethnic, low-income sample of infants and toddlers.* Public health nutrition, 2009. **12**(11): p. 2120-2128.

14. Pirkle, C.M., et al., *Food insecurity and nutritional biomarkers in relation to stature in Inuit children from Nunavik.* Canadian Journal of Public Health, 2014. **105**(4): p. e233-e238.

15. Shen, X., et al., *Food insecurity and malnutrition in Chinese elementary school students.* British Journal of Nutrition, 2015. **114**(6): p. 952-958.

16. Skalicky, A., et al., *Child food insecurity and iron deficiency anemia in low-income infants and toddlers in the United States.* Maternal and child health journal, 2006. **10**: p. 177-185.

17. Tiku, Y.S., et al., *Does Anaemia have major public health importance in children aged 6–59 months in the Duggina Fanigo District of Wolaita zone, southern Ethiopia?* Annals of Nutrition and Metabolism, 2018. **72**(1): p. 3-11.

18. Schmeer, K.K. and B.A. Piperata, *Household food insecurity and child health.* Maternal & Child Nutrition, 2017. **13**(2): p. e12301.

19. Basirat, R., A. SalehiAbarGhoei, and A. Esmaillzadeh, *The association between household food insecurity and childhood obesity among Iranian school-aged children in Farokhshahr.* Koomesh, 2012. **13**(2): p. 254-263.

20. Benjamin-Neelon, S.E., C. Allen, and B. Neelon, *Household food security and infant adiposity.* Pediatrics, 2020. **146**(3).

21. Do, E.K., et al., *Sleep, food insecurity, and weight status: Findings from the family life, activity, sun, health, and eating study.* Childhood Obesity, 2021. **17**(2): p. 125-135.

22. Drennen, C.R., et al., *Food insecurity, health, and development in children under age four years.* Pediatrics, 2019. **144**(4).

23. Farzaneh, H., et al., *Household food insecurity and its related socioeconomic and nutritional factors among northwest Iranian high-school students.* Rawal medical journal, 2017. **42**(2): p. 239-239.

24. Gundersen, C., S. Garasky, and B.J. Lohman, *Food insecurity is not associated with childhood obesity as assessed using multiple measures of obesity.* The Journal of nutrition, 2009. **139**(6): p. 1173-1178.

25. Gundersen, C., et al., *Child-specific food insecurity and overweight are not associated in a sample of 10-to 15-year-old low-income youth.* The Journal of nutrition, 2008. **138**(2): p. 371-378.

26. Holben, D.H. and C.A. Taylor, *Food insecurity and its association with central obesity and other markers of metabolic syndrome among persons aged 12 to 18 years in the United States.* Journal of Osteopathic Medicine, 2015. **115**(9): p. 536-543.

27. Jafari, F., et al., *Household food insecurity is associated with abdominal but not general obesity among Iranian children.* BMC public health, 2017. **17**: p. 1-8.

28. Jasmin, B., et al., *The association of household food security, household characteristics and school environment with obesity status among off-reserve First Nations and Métis children and youth in Canada: Results from the 2012 Aboriginal Peoples Survey.* Health Promotion and Chronic Disease Prevention in Canada: Research, Policy and Practice, 2017. **37**(3): p. 77.

29. Kaur, J., M.M. Lamb, and C.L. Ogden, *The association between food insecurity and obesity in children—The National Health and Nutrition Examination Survey.* Journal of the Academy of Nutrition and Dietetics, 2015. **115**(5): p. 751-758.

30. Martin, K.S. and A.M. Ferris, *Food insecurity and gender are risk factors for obesity.* Journal of nutrition education and behavior, 2007. **39**(1): p. 31-36.

31. Metallinos-Katsaras, E., A. Must, and K. Gorman, *A longitudinal study of food insecurity on obesity in preschool children.* Journal of the Academy of Nutrition and Dietetics, 2012. **112**(12): p. 1949-1958.

32. Rose, D. and J.N. Bodor, *Household food insecurity and overweight status in young school children: results from the Early Childhood Longitudinal Study.* Pediatrics, 2006. **117**(2): p. 464-473.

33. Whitaker, R.C. and S.M. Orzol, *Obesity among US urban preschool children: relationships to race, ethnicity, and socioeconomic status.* Archives of pediatrics & adolescent medicine, 2006. **160**(6): p. 578-584.

34. Alaimo, K., C.M. Olson, and E.A. Frongillo, *Low family income and food insufficiency in relation to overweight in US children: is there a paradox?* Archives of pediatrics & adolescent medicine, 2001. **155**(10): p. 1161-1167.

35. Casey, P.H., et al., *The association of child and household food insecurity with childhood overweight status.* Pediatrics, 2006. **118**(5): p. e1406-e1413.

36. Dubois, L., et al., *Family food insufficiency is related to overweight among preschoolers’.* Social science & medicine, 2006. **63**(6): p. 1503-1516.

37. Swindle, T., et al., *Application of noninferiority tests to examine the food insecurity–obesity relationship in children.* Journal of hunger & environmental nutrition, 2018. **13**(2): p. 228-239.

38. Ahmadihoseini, A., et al., *The relationship between food insecurity and anthropometric measures at birth in low income households.* Iranian journal of pediatrics, 2019. **29**(4).

39. Betebo, B., et al., *Household food insecurity and its association with nutritional status of children 6–59 months of age in east Badawacho District, south Ethiopia.* Journal of environmental and public health, 2017. **2017**(1): p. 6373595.

40. Isanaka, S., et al., *Food Insecurity Is Highly Prevalent and Predicts Underweight but Not Overweight in Adults and School Children from Bogotá, Colombia, 3.* The Journal of nutrition, 2007. **137**(12): p. 2747-2755.

41. Naser, I.A., et al., *Association between household food insecurity and nutritional outcomes among children in Northeastern of Peninsular Malaysia.* Nutrition research and practice, 2014. **8**(3): p. 304-311.

42. Shahraki, S.H., et al., *Household food insecurity is associated with nutritional status among Iranian children.* Ecology of food and nutrition, 2016. **55**(5): p. 473-490.

43. Singh, A., A. Singh, and F. Ram, *Household food insecurity and nutritional status of children and women in Nepal.* Food and nutrition bulletin, 2014. **35**(1): p. 3-11.

44. Speirs, K.E., B.H. Fiese, and S.K.R.T.S.i. edu, *The relationship between food insecurity and BMI for preschool children.* Maternal and child health journal, 2016. **20**: p. 925-933.

45. Wolde, M., Y. Berhan, and A. Chala, *Determinants of underweight, stunting and wasting among schoolchildren.* BMC public health, 2015. **15**: p. 1-9.

46. Belayneh, M., E. Loha, and B. Lindtjørn, *Seasonal variation of household food insecurity and household dietary diversity on wasting and stunting among young children in a drought prone area in South Ethiopia: a cohort study.* Ecology of food and nutrition, 2021. **60**(1): p. 44-69.

47. Cook, J.T., et al., *Child food insecurity increases risks posed by household food insecurity to young children’s health.* The Journal of nutrition, 2006. **136**(4): p. 1073-1076.

48. Hagos, S., et al., *Spatial heterogeneity and risk factors for stunting among children under age five in Ethiopia: A Bayesian geo-statistical model.* PLoS One, 2017. **12**(2): p. e0170785.

49. Kabalo, B.Y. and B. Lindtjørn, *Seasonality and predictors of childhood stunting and wasting in drought-prone areas in Ethiopia: a cohort study.* BMJ open, 2022. **12**(11): p. e060692.

50. Koyratty, N., et al., *Growth and growth trajectory among infants in early life: contributions of food insecurity and water insecurity in rural Zimbabwe.* BMJ Nutrition, Prevention & Health, 2022. **5**(2): p. 332.

51. Mahmudiono, T., et al., *Household food insecurity as a predictor of stunted children and overweight/obese mothers (SCOWT) in urban Indonesia.* Nutrients, 2018. **10**(5): p. 535.

52. McDonald, C., et al., *Household food insecurity and dietary diversity as correlates of maternal and child undernutrition in rural Cambodia.* European Journal of Clinical Nutrition, 2015. **69**(2): p. 242-246.

53. Mutisya, M., et al., *Household food (in) security and nutritional status of urban poor children aged 6 to 23 months in Kenya.* BMC public health, 2015. **15**: p. 1-10.

54. Na, M., et al., *Maternal nutritional status mediates the linkage between household food insecurity and mid-infancy size in rural Bangladesh.* British Journal of Nutrition, 2020. **123**(12): p. 1415-1425.

55. Namirembe, G., et al., *Child stunting starts in utero: Growth trajectories and determinants in Ugandan infants.* Maternal & child nutrition, 2022. **18**(3): p. e13359.

56. Nkurunziza, S., et al., *Determinants of stunting and severe stunting among Burundian children aged 6-23 months: evidence from a national cross-sectional household survey, 2014.* BMC pediatrics, 2017. **17**: p. 1-14.

57. Shinsugi, C., et al., *Factors Associated with Stunting Among Children According to the Level of Food Insecurity in the Household: A Cross-Sectional Study in a Rural Community of Southeastern Kenya........................................... Mwatasa Changoma, and Satoshi Kaneko*, in *Food Security and Child Malnutrition*. 2016, Apple Academic Press. p. 135-154.

58. Angelopoulou, M.V., et al., *Association of food insecurity with early childhood caries.* Journal of public health dentistry, 2019. **79**(2): p. 102-108.

59. Bae, J.-H. and B.W.O. Obounou, *Presence of dental caries is associated with food insecurity and frequency of breakfast consumption in Korean children and adolescents.* Preventive nutrition and food science, 2018. **23**(2): p. 94.

60. Bahanan, L., et al., *The association between food insecurity, diet quality, and untreated caries among US children.* The Journal of the American Dental Association, 2021. **152**(8): p. 613-621.

61. Braunstein, N.S., *Diet, Food Insecurity and Dental Caries Prevalence and Severity in Children Ages 2--11*. 2008: ProQuest.

62. Chi, D.L., et al., *Socioeconomic status, food security, and dental caries in US children: mediation analyses of data from the National Health and Nutrition Examination Survey, 2007–2008.* American journal of public health, 2014. **104**(5): p. 860-864.

63. Ferreira, F.M., et al., *How much does household food insecurity explain income inequalities in untreated dental caries?* International journal of paediatric dentistry, 2019. **29**(3): p. 325-331.

64. Frazao, P., et al., *Food insecurity and dental caries in schoolchildren: a cross‐sectional survey in the western B razilian A mazon.* European journal of oral sciences, 2014. **122**(3): p. 210-215.

65. Jackson, D.B. and A. Testa, *Household food insecurity and children's oral health: findings from the 2016–2018 National Survey of Children's Health.* Journal of public health dentistry, 2021. **81**(2): p. 150-161.

66. Santin, G.C., et al., *Association between untreated dental caries and household food insecurity in schoolchildren.* Ciencia & saude coletiva, 2016. **21**: p. 573-584.

67. Tsai, W.T. and H.P. Lawrence, *Association between psychosocial determinants of adverse childhood experiences and severe early childhood caries among First Nations children.* International Journal of Paediatric Dentistry, 2022. **32**(3): p. 352-366.

68. Alaimo, K., C.M. Olson, and E.A. Frongillo, *Family food insufficiency, but not low family income, is positively associated with dysthymia and suicide symptoms in adolescents.* The Journal of nutrition, 2002. **132**(4): p. 719-725.

69. Almansour, A.M. and S. Siziya, *Suicidal ideation and associated factors among school going adolescents in Swaziland.* African health sciences, 2017. **17**(4): p. 1172-1177.

70. Altangerel, U., J.-C. Liou, and P.-M. Yeh, *Prevalence and predictors of suicidal behavior among Mongolian high school students.* Community mental health journal, 2014. **50**: p. 362-372.

71. Arat, G., *The link between nutrition and mental health in sub-Saharan African adolescents: findings from the global school-based health survey.* Global social welfare, 2017. **4**: p. 31-40.

72. Asante, K.O., et al., *The prevalence and correlates of suicidal behaviours (ideation, plan and attempt) among adolescents in senior high schools in Ghana.* SSM-population health, 2017. **3**: p. 427-434.

73. Dema, T., et al., *Suicidal ideation and attempt among school going adolescents in Bhutan–a secondary analysis of a global school-based student health survey in Bhutan 2016.* BMC public health, 2019. **19**: p. 1-12.

74. Kwangu, M., et al., *Adolescents attending school in the Philippines and suicidal ideation.* AGlobal VIEW ON SUICIDAL IDEATION AMONG ADOLESCENTS, 2017: p. 51.

75. Mazaba, M.L., et al., *Suicidal ideation among Arab adolescents in school in the west bank.* AGlobal VIEW ON SUICIDAL IDEATION AMONG ADOLESCENTS, 2017: p. 163.

76. Mazaba, M.L., et al., *Suicidal ideation in Fiji: Prevalence and its correlates among school-going adolescents in a global school health-based survey.* International Public Health Journal, 2017. **9**(4).

77. Mazaba, M.L., et al., *Suicidal ideation among adolescents attending school in Kuwait.* AGlobal VIEW ON SUICIDAL IDEATION AMONG ADOLESCENTS, 2017: p. 141.

78. Mazaba, M.L., et al., *Suicidal ideation in Vietnam.* AGlobal VIEW ON SUICIDAL IDEATION AMONG ADOLESCENTS, 2017: p. 183.

79. Mulenga, D., et al., *Suicidal ideation among school‐going adolescents in Iraq.* Suicide, 2017. **9**(4): p. 123-130.

80. Mulenga, D., et al., *Suicidal ideation among adolescents in school in the gaza strip.* AGlobal VIEW ON SUICIDAL IDEATION AMONG ADOLESCENTS, 2017: p. 33.

81. Mulenga, D., et al., *Suicidal ideation among in-school adolescents in Trinidad and Tobago.* 2017.

82. Mwenya Kwangu, B., *Suicidal ideation prevalence and its associated factors among school-going adolescents in Pakistan.* International Public Health Journal, 2017. **9**(4): p. 401-406.

83. Mwenya Kwangu, B., *Prevalence and factors for suicide ideation among adolescents attending school in Ghana.* International Public Health Journal, 2017. **9**(4): p. 365-372.

84. Nyundo, A., et al., *Factors associated with depressive symptoms and suicidal ideation and behaviours amongst sub‐Saharan African adolescents aged 10‐19 years: cross‐sectional study.* Tropical Medicine & International Health, 2020. **25**(1): p. 54-69.

85. Pandey, A.R., et al., *Factors associated with suicidal ideation and suicidal attempts among adolescent students in Nepal: Findings from Global School-based Students Health Survey.* PloS one, 2019. **14**(4): p. e0210383.

86. Pengpid, S. and K. Peltzer, *Single and multiple suicide attempts: prevalence and correlates in school-going adolescents in Liberia in 2017.* Psychology research and behavior management, 2020: p. 1159-1164.

87. Romo, M.L., V. Abril-Ulloa, and E.A. Kelvin, *The relationship between hunger and mental health outcomes among school-going Ecuadorian adolescents.* Social Psychiatry and Psychiatric Epidemiology, 2016. **51**: p. 827-837.

88. Shayo, F.K. and P.S. Lawala, *Does food insecurity link to suicidal behaviors among in-school adolescents? Findings from the low-income country of sub-Saharan Africa.* BMC psychiatry, 2019. **19**: p. 1-8.

89. Siziya, S., et al., *Suicidal ideation, adolescents and school in Samoa*. 2017. p. 111-122.

90. Siziya, S., et al., *Suicidal ideation in Jamaica: Prevalence and its correlates among school-going adolescents in a global school health-based survey.* International Public Health Journal, 2017. **9**(4).

91. Teevale, T., et al., *Risk and protective factors for suicidal behaviors among Pacific youth in New Zealand.* Crisis, 2016.

92. Ziaei, R., et al., *Suicidal ideation and its correlates among high school students in Iran: a cross-sectional study.* BMC psychiatry, 2017. **17**: p. 1-7.

93. Mulenga, D., et al., *Suicidal ideation among in-school adolescents in Morocco*. 2017. p. 175-182.

94. Quarshie, E.N.-B. and J. Andoh-Arthur, *Suicide attempts among 1,437 adolescents aged 12–17 years attending junior high schools in Ghana.* Crisis, 2020.

95. Njunju, E.M., Kwangu, M., Siziya, S., Mulenga, D., Mazaba, M.L., *Suicidal ideation among in-school adolescents in Myanmar.* 2017.

96. Black, M.M., et al., *WIC participation and attenuation of stress-related child health risks of household food insecurity and caregiver depressive symptoms.* Archives of pediatrics & adolescent medicine, 2012. **166**(5): p. 444-451.

97. Finch, J.E., et al., *Measuring and understanding social-emotional behaviors in preschoolers from rural Pakistan.* PloS one, 2018. **13**(11): p. e0207807.

98. Gill, M., M. Koleilat, and S.E. Whaley, *The impact of food insecurity on the home emotional environment among low-income mothers of young children.* Maternal and child health journal, 2018. **22**: p. 1146-1153.

99. Hernandez, D.C. and A. Jacknowitz, *Transient, but not persistent, adult food insecurity influences toddler development.* The Journal of nutrition, 2009. **139**(8): p. 1517-1524.

100. Hobbs, S. and C. King, *The unequal impact of food insecurity on cognitive and behavioral outcomes among 5-year-old urban children.* Journal of Nutrition Education and Behavior, 2018. **50**(7): p. 687-694.

101. Huang, Y., S. Potochnick, and C.M. Heflin, *Household food insecurity and early childhood health and cognitive development among children of immigrants.* Journal of Family Issues, 2018. **39**(6): p. 1465-1497.

102. Johnson, A.D. and A.J. Markowitz, *Associations between household food insecurity in early childhood and children's kindergarten skills.* Child Development, 2018. **89**(2): p. e1-e17.

103. Mickens, A.J., *The impact of poverty on math readiness*. 2019, The University of Arizona.

104. Milner, E.M., et al., *Timing, intensity, and duration of household food insecurity are associated with early childhood development in K enya.* Maternal & Child Nutrition, 2018. **14**(2): p. e12543.

105. Nagata, J.M., et al., *Food insecurity is associated with maternal depression and child pervasive developmental symptoms in low-income Latino households.* Journal of hunger & environmental nutrition, 2019. **14**(4): p. 526-539.

106. Obradović, J., et al., *Maternal scaffolding and home stimulation: Key mediators of early intervention effects on children’s cognitive development.* Developmental psychology, 2016. **52**(9): p. 1409.

107. Rose-Jacobs, R., et al., *Household food insecurity: associations with at-risk infant and toddler development.* Pediatrics, 2008. **121**(1): p. 65-72.

108. Whitaker, R.C., S.M. Phillips, and S.M. Orzol, *Food insecurity and the risks of depression and anxiety in mothers and behavior problems in their preschool-aged children.* Pediatrics, 2006. **118**(3): p. e859-e868.

109. Belachew, A. and T. Tewabe, *Under-five anemia and its associated factors with dietary diversity, food security, stunted, and deworming in Ethiopia: systematic review and meta-analysis.* Systematic reviews, 2020. **9**: p. 1-9.

110. Derakhshandeh-Rishehri, S.-M., Z. Hassanzadeh-Rostami, and S. Faghih, *Association between Food Insecurity and Weight Disorders of Children and Adolescents in Iranian Population: A Systematic Review and Meta-Analysis.* Journal of Nutrition and Food Security, 2022. **7**(2): p. 237-247.

111. Drumond, V.Z., et al., *Burden of dental caries in individuals experiencing food insecurity: a systematic review and meta-analysis.* Nutrition Reviews, 2023. **81**(12): p. 1525-1555.

112. Eskandari, F., et al., *A mixed‐method systematic review and meta‐analysis of the influences of food environments and food insecurity on obesity in high‐income countries.* Food Science & Nutrition, 2022. **10**(11): p. 3689-3723.

113. Kaggwa, M.M., et al., *The global burden of suicidal behavior among people experiencing food insecurity: A systematic review and meta-analysis.* Journal of Affective Disorders, 2023.

114. Moradi, S., et al., *Food insecurity and anaemia risk: a systematic review and meta-analysis.* Public health nutrition, 2018. **21**(16): p. 3067-3079.

115. Moradi, S., et al., *Food insecurity and the risk of undernutrition complications among children and adolescents: a systematic review and meta-analysis.* Nutrition, 2019. **62**: p. 52-60.

116. de Oliveira, K.H.D., et al., *Household food insecurity and early childhood development: Systematic review and meta‐analysis.* Maternal & child nutrition, 2020. **16**(3): p. e12967.

117. Patriota, É.S., et al., *Association between household food insecurity and stunting in children aged 0− 59 months: Systematic review and meta‐analysis of cohort studies.* Maternal & Child Nutrition, 2024. **20**(2): p. e13609.

118. Pourmotabbed, A., et al., *The relationship between food insecurity and risk of overweight or obesity in under 18 years individuals: A systematic review and meta-analysis.* International Journal of Preventive Medicine, 2020. **11**(1): p. 158.

119. Sabbagh, S., et al., *Food insecurity and dental caries prevalence in children and adolescents: A systematic review and meta‐analysis.* International Journal of Paediatric Dentistry, 2023. **33**(4): p. 346-363.

120. Zhou, C., et al., *Food insecurity increases the risk of overweight and chronic diseases in adolescents: a systematic review and meta-analysis.* Food Science and Human Wellness, 2023. **12**(6): p. 1937-1947.
